# Recent developments in d-α-tocopheryl polyethylene glycol-succinate-based nanomedicine for cancer therapy

**DOI:** 10.1080/10717544.2017.1406561

**Published:** 2017-11-28

**Authors:** Songwei Tan, Chenming Zou, Wei Zhang, Mingxing Yin, Xueqin Gao, Qing Tang

**Affiliations:** a School of Pharmacy, Tongji Medical College, Huazhong University of Science and Technology, Wuhan, China;; b Department of Pharmacy, Tongji Hospital, Tongji Medical School, Huazhong University of Science and Technology, Wuhan, China;; c Department of Integrated Chinese and Western Medicine, Union Hospital, Tongji Medical College, Huazhong University of Science and Technology, Wuhan, China;

**Keywords:** d-α-tocopheryl polyethylene glycol succinate (TPGS), cancer, nanomedicine, multidrug-resistant, drug delivery system

## Abstract

Cancer remains an obstacle to be surmounted by humans. As an FDA-approved biocompatible drug excipient, d-α-tocopheryl polyethylene glycol succinate (TPGS) has been widely applied in drug delivery system (DDS). Along with in-depth analyses of TPGS-based DDS, increasingly attractive results have revealed that TPGS is able to act not only as a simple drug carrier but also as an assistant molecule with various bio-functions to improve anticancer efficacy. In this review, recent advances in TPGS-based DDS are summarized. TPGS can inhibit P-glycoprotein, enhance drug absorption, induce mitochondrial-associated apoptosis or other apoptotic pathways, promote drug penetration and tumor accumulation, and even inhibit tumor metastasis. As a result, many formulations, by using original TPGS, TPGS-drug conjugates or TPGS copolymers, were prepared, and as expected, an enhanced therapeutic effect was achieved in different tumor models, especially in multidrug resistant and metastatic tumors. Although the mechanisms by which TPGS participates in such functions are not yet very clear, considering its effectiveness in tumor treatment, TPGS-based DDS appears to be one of the best candidates for future clinical applications.

## Introduction

Nanotechnology has been explored in medical research for many years. Because of the unique properties of nano-sized drug delivery system (DDS), significant progress has been reported in areas of cancer treatment (Mura et al., 2013; Masood, 2016; Shi et al., 2017). However, several challenges persist for the clinical application of nanomedicine, for example, the drug carriers (Wicki et al., 2015). To achieve enhanced therapeutic efficacy, multi-functional DDSs are typically developed that have a complex structure that may obstruct further pre-clinical evaluations. Therefore, bio-compatibility and bio-safety are becoming areas of increasing focus in the design of a new DDS. Thus, taking advantage of the bio-function of drug carriers themselves may be a potential way to develop a simple but multi-functional DDS. For example, hyaluronic acid (HA) is capable of binding to CD 44 and can be used for tumor-targeted drug delivery carrier without further targeting modification, and poloxamers and d-α-tocopheryl polyethylene glycol succinate (TPGS) can inhibit the activity of P-gp and thus are widely used as the component of DDS to stabilize the formulation and overcome multidrug resistance (MDR) in tumors (Saneja et al., 2014; Rao et al., 2016; Muddineti et al., 2017).

TPGS, the water-soluble derivative of natural vitamin E (VE), is a typical multi-functional material and has attracted increasing attention in nanomedicine during the past few years. TPGS is the esterification product of VE succinate with polyethylene glycol (PEG). When the molecular weight (Mw) of PEG is 1000, the product is denoted TPGS1000, or simply TPGS, which is the most used form in TPGS-based nanomedicine. If the Mw of PEG is different, the name should be denoted as TPGS*x* (where *x* is the Mw of PEG), for example, TPGS450 (M_PEG_ = 450) and TPGS2K (M_PEG_ = 2000). Changes in the PEG are also related to the physicochemical/biological properties, including the critical micelle concentration (CMC), hydrophile-lipophile balance (HLB) value, P-gp inhibiting activity and even the circulation time after i.v. administration.

**Figure 1.**
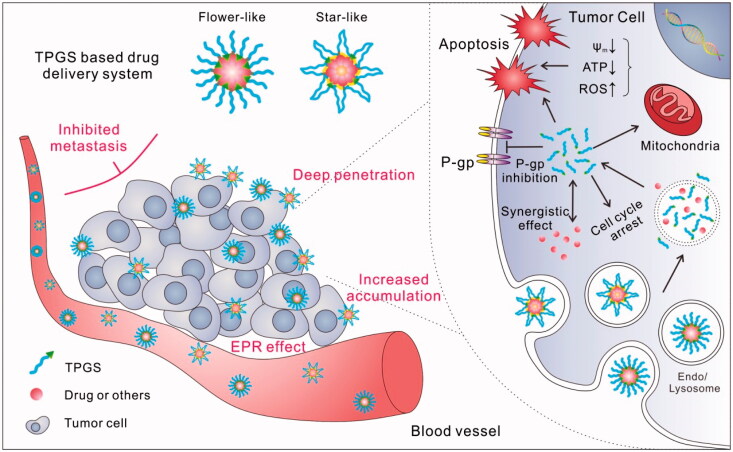
Scheme of potential bio-function of TPGS-based DDS for the treatment of cancer.

The benefits of exploring TPGS in nanomedicine include the following: (1) safety. TPGS is an FDA- and CFDA-approved pharmaceutical excipient, with an oral LD_50_ >7 g/kg in adult rats. (2) Universal. As a nonionic surfactant, TPGS can be applied in many different DDSs, such as micelles, liposomes, and nanoparticles (NPs). (3) P-gp inhibition. Although many nonionic surfactants, such as Pluronic^®^ and Tween, can inhibit the activity of P-gp, TPGS has been reported as the most effective one among them. Due to its special properties, TPGS can be used as an oral absorption enhancer and an agent to overcome MDR in tumors. (4) Tumor cell cytotoxicity. TPGS has also been found to be cytotoxic to tumor cells, likely due to reactive oxygen species (ROS) generation and mitochondrial-associated apoptosis. Previously published reviews had summarized the basic properties and applications of TPGS, including P-gp inhibitor, solubilizer, absorption/permeation enhancer, and anticancer activity in DDS (Zhang et al., 2012; Guo et al., 2013; Duhem et al., 2014). In this review, we first discuss new bio-functions and related mechanisms of TPGS revealed in recent years (such as tumor permeation and accumulation enhancer, cell uptake enhancer, mitochondrial-associated apoptotic pathways inducer, and metastasis inhibitor, Figure 1) and then listed some typical and new design examples of TPGS-based anticancer DDSs according to the detail formulation, including original TPGS (without any modifications) containing DDS, TPGS-drug conjugates (prodrugs), and TPGS-based copolymers. The TPGS-based anticancer formulations were also summarized in Table S1. We expect that this review will help to clarify recent advances in the use of TPGS for the treatment of cancer and broaden the horizon of its future applications.

## Bio-functions of TPGS

### P-gp inhibitor and absorption/uptake enhancer

The most fascinating feature of the application of TPGS for cancer treatment is its P-gp-inhibiting ability. Based on this property, many TPGS-containing DDSs have been designed and acted as a high-efficiency reversal agent for P-gp-mediated MDR cancer. Although the ability of TPGS to inhibit P-gp was reported in 1999, the mechanism was revealed nearly 10 years later by Collnot et al. as a reduction of P-gp ATPase activity by steric blocking of the binding site and/or allosteric modulation of P-gp by the direct interaction between TPGS and allosteric sites in the efflux pump (Dintaman & Silverman, 1999; Collnot et al., 2007; Collnot et al., 2010). Other studies showed that TPGS only inhibits the bio-function of P-gp but does not influence the P-gp expression level of MDR cells, which was thought to be closely related to its VE moiety (Tang et al., 2013; Qiu et al., 2014; Wang et al., 2015a; Qiao et al., 2017). Collnot et al. also examined the ability of TPGS*x* (where *x* varies from 20 to 6000) to inhibit P-gp and suggested that the optimal PEG chain length was between 1100 and 1500 Da. Thus, commercial TPGS (e.g. TPGS1000) was commonly adopted for its convenience and outstanding P-gp inhibition ability. Moreover, TPGS2K was found to have a similar ability to overcome MDR (Qiu et al., 2014). Therefore, it was also applied in some cases due to its long PEG segment, which may introduce some advantages compared with TPGS1000.

Because of the P-gp inhibition ability of TPGS, either the oral bioavailability or the drug concentration in MDR cancer cells of anticancer agents could be greatly improved and thus enhanced treatment efficacy (Guo et al., 2013; Choudhury et al., 2017). An interesting phenomenon is that even in non-MDR cancer cells, the drug amount was still increased by TPGS-containing DDS, as compared with PEG-modified DDS with a similar structure and particle size (Bernabeu et al., 2014; Bernabeu et al., 2016; Cheng et al., 2017). A possible explanation is that the VE portion of TPGS may act as a ligand that interacts with some receptors in the cancer cell membrane and thus induces receptor-mediated endocytosis to facilitate drug absorption (Takada & Suzuki, 2010; Cardenas & Ghosh, 2013).

### Enhanced permeation and accumulation

**Figure 2.**
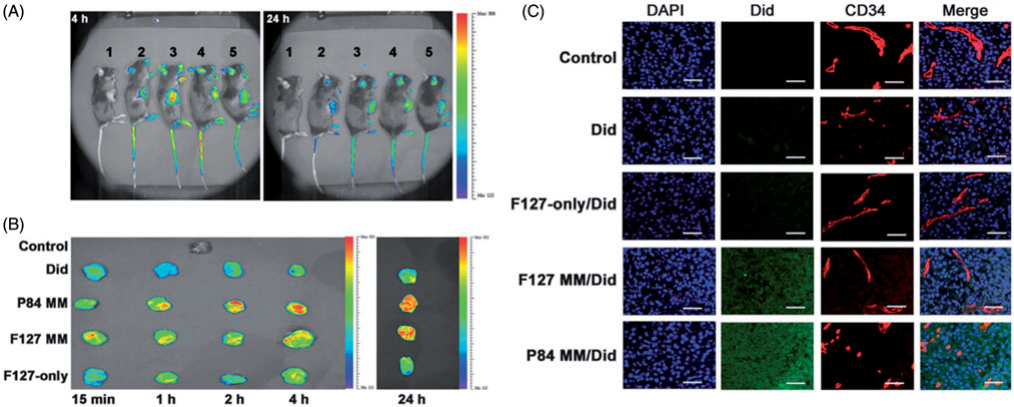
TPGS improves drug accumulation and penetration in tumors. (A) *In vivo* imaging, (B) *Ex vivo* imaging of excised tumors and (C) Intratumoral distributions of Did-loaded micelles in B16F10 tumor bearing mice. 1, Saline; 2, Did; 3, P84-TPGS mixed micelles; 4, F127 -TPGS mixed micelles; 5, F127 micelles (Cao et al., 2016).

TPGS can be used as a drug permeation enhancer to promote penetration through skin, alimentary canal walls or cornea (Fan et al., 2013; Duan et al., 2015; Pham & Cho, 2017). A recent work has shown that TPGS may increase drug permeation in solid tumors by using a MDR KBv tumor spheroid model (Wang et al., 2015a). Compared with Rhodamine-123 and TPGS-free micelles, the TPGS hybrid micelles showed a more than 10-fold higher permeation depth. The authors proposed that TPGS played a key role in the tumor spheroid permeation and accumulation. Similar results were observed using the TPGS hybrid Genexol-PM system in A549 tumor spheroids (Mohapatra et al., 2015). Cao et al. (2016) also reported that TPGS-containing Pluronic micelles could diffuse/penetrate into the core of B16F10 cell spheroids more effectively and remain there much longer than pure Pluronic micelles, indicating that most of the micelles were retained intracellularly rather than being exocytosed or degraded. Results obtained for tumor xenografts have also confirmed that TPGS can enhance the tumor accumulation of micelles in terms of time, space, and amount (faster, deeper, and more, Figure 2). The strong penetration ability is thought to be caused by interstitial extracellular matrix diffusion, but not the transcytosis pathway.

TPGS can even help the drug traverse the blood brain barrier (BBB). Compared with Taxotere, TPGS micelles can improve the docetaxel (DTX) concentration in rats brain 2.5-fold after i.v. administration for 2 h (Muthu et al., 2012). A recent study has also revealed that TPGS/F127 mixed micelles can deliver DiR to the brain more effectively than F127 micelles, with a 3.6-fold greater intensity observed at 2 h after i.v. injection in rats despite similar sizes of approximately 20 nm (Meng et al., 2017). This finding may be related to the enhanced cell uptake in rat brain endothelial cells, which play a key role in the barrier properties of the BBB due to nonspecific absorption *via* an absorption-mediated endocytic pathway and P-gp inhibition of TPGS.

### Mitochondrial-associated apoptotic pathways and anticancer properties

VE is described as a type of antioxidant; however, this bio-function is challenged because the concentration must be sufficiently high to meet this task (Brigelius-Flohé & Galli, 2010). Interestingly, some researchers have reported the presence of mitochondrial-associated apoptotic pathways in cancer cells induced by ROS generation and/or energy metabolism inhibition in VE derivatives (Neuzil et al., 2007a,b; Dong et al., 2009; Cheng et al., 2013). A previous study showed that TPGS could suppress the growth of human H460 and A549 lung carcinoma cells *in vitro* (20–50 μmol/L or 30–75 mg/L) and A549 xenografted tumors in nude mice (250 μL, 40 mmol/L) due to its ability to generate ROS and induce apoptosis (Youk et al., 2005). Another study also reported the cytotoxicity of TPGS2K micelles (25 mg/L) against MCF-7 cells *in vitro* (Mi et al., 2011). The molecular mechanism of the cytotoxicity of TPGS was investigated by Neophytou et al. (2014) in breast cancer MCF-7 and MDA-MB-231 cells. The results showed that TPGS could inhibit the phosphorylation of protein kinase B (AKT), down-regulate Survivin and Bcl-2, and ultimately induce apoptosis. Another effect was increased protein levels of P21 and P27Kip1, which promoted cell cycle arrest at G1 phase. However, these phenomena were hardly observed in normal MCF-10 A and MCF-12 F cells (non-tumorigenic but immortalized). Another work also demonstrated that TPGS containing drug-free NPs was cytotoxic to A549 cells but nontoxic to human hepatic cells (L02) (Abbad et al., 2015). Recently, it has been demonstrated that TPGS was capable of elevating intracellular ROS production, lowering mitochondria membrane potential and down-regulating ATP levels in drug-resistant MCF-7/ADR cell lines, and these capabilities were further strengthened in the presence of the pH-sensitive TPGS-DOX prodrug, HA-TPGS copolymers, and other TPGS-containing DDS (Qiu et al., 2014; Su et al., 2014; Wang et al., 2015a; Bao et al., 2016). A possible explanation for these effects was a strong interaction between TPGS and the mitochondrial respiratory complex II, reducing its activity and thus resulting in the leakage of electrons and subsequent generation of ROS *via* their combination with molecular oxygen (Su et al., 2014; Kai et al., 2016). When combined with tocopheryl succinate (TOS), the blank TPGS2K/TOS mixed micelles could even exhibit equivalent anticancer efficacy to free doxorubicin (DOX) against CT26 and MCF-7 tumor-bearing mice (Danhier et al., 2014). In anticancer drug and TPGS combinations, a strong synergistic effect may be helpful for increasing the anticancer ability, especially for overcoming MDR of cancer.

### Metastasis inhibition

Another interesting and valuable bio-function of TPGS is its potential to inhibit the metastasis of cancer. Li’ group first confirmed that TPGS containing blank lipid NPs (BLNs) had efficient anti-metastatic activity in a wound healing assay and that this effect was dose-dependent (Xu et al., 2013). In spontaneous lung metastasis, a decrease in the number of metastatic nodules was observed in the BLNs group compared with the drug silibinin (SIL). Kutty et al., (2015) and our unpublished data also showed that TPGS had comparable capabilities for inhibiting the migration of MDA-MB-231 and B16F10 cells *in vitro* in a wound healing assay. A possible explanation was that TPGS could inhibit the expression of some metastasis-related proteins, such as matrix metalloproteinase 9 (MMP9), Snail. Likely owing to the metastasis inhibition properties of TPGS and/or the synergistic effect of the incorporated drug and TPGS, some TPGS-based DDS showed significant metastasis inhibitory rates by down-regulating metastasis-promoting proteins, including MMP9, Snail, Twist, anti-urokinase-type plasminogen activator, vascular endothelial growth factor, interleukin-1b and insulin-like growth factor 1, which would greatly expand the range of applications of TPGS for the treatment of tumors (Shen et al., 2013; Xu et al., 2013; Xu et al., 2014; Kutty et al., 2015; Bao et al., 2016).

## TPGS containing nano-sized DDS

As a surfactant, TPGS has an amphiphilic structure containing a long hydrophilic PEG head and a short lipophilic alkyl tail. The HLB value is 13.2, and the CMC of TPGS micelles is 0.02 wt% (measured by the surface tension method) or 0.02 mM (∼0.003 wt%, measured by the pyrene fluorescent probe method). Thus, TPGS is widely used as a solubilizer and emulsifier in DDS to form various formulations.

### Micelles or mixed micelles

TPGS can be either used alone or mixed with various materials, including other surfactants, phospholipids and polymers to solubilize hydrophobic drugs, increase permeability, and inhibit efflux to promote oral absorption and anticancer efficiency *via* oral administration. Due to its P-gp inhibition ability, TPGS can be used either as a drug carrier directly or added to the DDS to overcome the MDR of the cells. For example, Hao et al., (2015) prepared DOX-loaded TPGS2K micelles, which showed enhanced cellular uptake and cytotoxicity against MCF-7/MDR cells, as well as higher anticancer activity compared with free DOX. Qiu et al., (2014) described a pH-sensitive HA-g-poly(l-histidine) (HA-PHis)/TPGS2K mixed micelles to achieve pH-dependent drug release and inhibit P-gp-mediated drug efflux. The addition of TPGS2K could promote drug accumulation in MDR cells and thus reverse MDR in MCF-7/ADR cells. In addition, TPGS2K was also able to promote the tumor drug amount, which was thought to be related to the increased circulation time.

In addition to comparative simple micelles, TPGS-based multifunctional micelle systems have also been reported. Feng’s group developed modified TPGS micelles carrying both the antibody drug cetuximab and the chemotherapeutic drug DTX to treat triple-negative breast cancers (Kutty & Feng; 2013; Kutty et al., 2015). The micelles increased the uptake and cytotoxicity of triple-negative breast cancer cells (MDA-MB-468 and MDA-MB-231) with much higher efficiency and realized tumor growth inhibition compared with Taxotere and non-targeting micelles in tumor-bearing mice. Anti-angiogenesis and metastasis inhibition were also observed in an *ex vivo* investigation.

**Figure 3.**
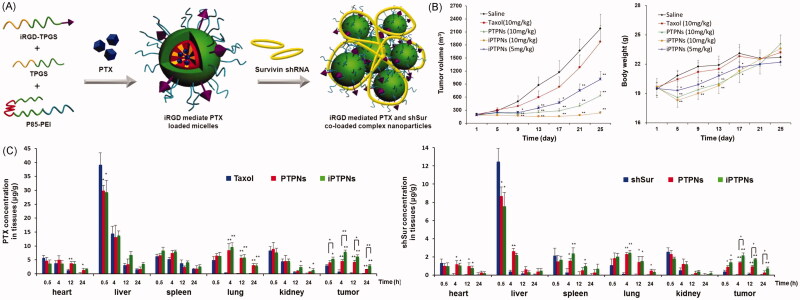
Mixed micelle-based complex NPs for the co-delivery of chemo-drug and shRNA. (A) Schematic illustration of NP preparation. (B) Anticancer effects and body weight changes of different treatments in A549/T bearing nude mice. (C) *In vivo* biodistribution of different formulations of PTX or shSur in different formulation at 0.5, 4, 12, and 24 h in A549/T bearing nude mice (Shen et al., 2014a).

Li’s group developed a mixed micelle-based complex NPs to co-deliver chemotherapeutic drug [paclitaxel (PTX) or sorafenib (SF)] and shRNA (Twist or survivin shRNA) (Figure 3) (Shen et al., 2013; Shen et al., 2014a,b). This system showed effective cell uptake, transfection and cytotoxicity against 4T1, BEL-7402, and BEL-7402/5Fu cells *in vitro*, a superior biodistribution in a tumor-bearing nude mouse model, good anticancer efficacy and the ability to reduce pulmonary metastasis in a 4T1 pulmonary metastatic mouse model. They further induced TPGS-iRGD in this system to achieve active targeting of the tumor tissue and to overcome MDR in A549/T cells. This system clearly showed superior performance *in vitro* and *in vivo* against A549 and A549/T cells. The tumor volume of complex NPs group was approximately 1/8 of that in the Taxol group.

### TPGS-containing lipid DDS

TPGS can be added to lipid DDS such as liposomes, solid lipid NPs (SLNs), and self-microemulsifying DDS (SMEDDS) to increase solubility, anticancer efficacy, MDR-inhibiting ability, oral absorption and even to act as a bridge to achieve targeted delivery. Lv’s group prepared TPGS containing liposome with TPGS-triphenylphosphine conjugates to achieve mitochondrial targeting of liposomes (Zhou et al., 2013; Shi et al., 2015). TPGS not only benefited to the long circulation time but also increased liposome uptake by MDR A549/cDDP cells and further endocytosed by the mitochondria, which induced the mitochondrial signaling pathway leading to apoptosis. Thus, the greater anticancer activity was observed in a xenografted tumor model than Taxol and PTX liposomes. Such system could also target vasculogenic mimicry (VM) channels and induce cytotoxic injury and apoptosis to prevent recurrence caused by VM channels. In addition, Assanhou et al., (2015) reported a direct mitochondrial-targeting phenomenon using liposomes functionalized by TPGS. Xu et al., (2013) developed TPGS containing SIL-loaded SLNs to inhibit the metastasis of breast cancer. The SLNs could reach the tumor tissue efficiently. By down-regulating MMP9 and Snail, the SLNs demonstrated strong migration inhibition effects against MDA-MB-231 cells both *in vitro* and *in vivo*. Cho also prepared TPGS-modified SLNs to enhance intestinal absorption of DTX, which demonstrated a higher relative oral bioavailability of DTX than Tween 80-emulsified SLNs (Cho et al., 2014).

SMEDDS can increase the solubility and oral bioavailability of a drug, and thus, it is widely applied in oral preparations. DOX-SMEDDS was developed recently by lipidizing DOX as a Dox-aerosol OT (AOT) ion pair complex (Benival & Devarajan, 2015). It formed a microemulsion after hydration with the size of ∼200 nm and showed 4.2-fold enhanced oral bioavailability compared with DOX solution. This SMEDDS could inhibit the growth of fibrosarcoma in mice with reduced heart and kidney toxicity. Similarly, Valicherla et al. (2016) reported results for DTX-SMEDDS with 3.2-fold greater oral bioavailability and a higher tumor accumulation than Taxotere.

### TPGS-stabilized nanocrystals (NCs), nanosuspensions (NSs), and NPs

Drug NCs and NSs are useful preparations for solving some of the problems related to hydrophobic drugs, such as their poor solubility, dissolution, and bioavailability. TPGS were also applied as a stabilizer in this system and exhibited some attractive properties. For example, the solubility of curcumin (CUR, 0.6 g/mL) was increased to 260 g/mL in NC form (Shin et al., 2016). For CPT NS, the solubility increased 16 times, and the *in vitro* and *in vivo* performance were all improved compared to CPT solution in MCF-7 cell line (Tang et al., 2014). Liu et al. (2010, 2016b) developed TPGS-PTX NCs and found that they could accumulate in MDR tumors more effectively and overcome the MDR of NCI/ADR-RES cells both *in vitro* and *in vivo*. In another case, the LD_50_ of TPGS-PTX NSs was 2.4 times greater than Taxol (Gao et al., 2013; Gao et al., 2014). Using MDR H460/RT xenograft tumor model, it was found to be five-fold more effective than the mixed solution of PTX and TPGS. Li’s group prepared directed self-assembled NPs (SCNs) of SF and CUR by using TPGS as the stabilizer (Cao et al., 2015). Compared with SF, CUR, and their physical mixture, SCNs showed increased cytotoxicity toward BEL-7402 cells and HepG2 cells and enhanced antiangiogenesis activities *in vitro*. After oral administration, the concentration of SF and CUR in tumors was enhanced by 4.2- and 5.9-fold for SCNs compared with the free drug suspension. As a result, SCNs displayed the strongest tumor growth inhibition in a BEL-7402 cell xenograft tumor model.

TPGS can be further used to increase the stability and drug-loading ability and also to improve the *in vitro* and *in vivo* performance of both inorganic and organic NPs. For example, TPGS helped to improve the water-solubility of NaYbF_4_:Er upconversion NPs or gold clusters, and to co-load chemo-drug (DOX or DTX), thus functioning as a multi-functional system for imaging probe and overcoming the MDR of MCF-7/ARD or MDA-MB-231-luc cells. (Muthu et al., 2015; Tian et al., 2015). In another case, PTX-loaded bovine serum albumin NPs showed doubled IC_50_ value compared with Taxol in both MCF-7 and MCF-7/ADR cells. However, with the addition of TPGS, the IC_50_ value became comparable to that observed with Taxol in MCF-7 cells and demonstrated a dramatic decrease of more than five-fold compared with Taxol in MCF-7/ADR cells (Chen et al., 2016). Han et al. developed lipid-capped mesoporous silica NPs (MSNs). The TPGS-containing lipid layer was fixed on the MSN surface *via* disulfide bonds so the loaded DOX could achieve pH- and redox-dual-responsible release, which resulted in higher drug accumulation and cytotoxicity in both MCF-7 and MCF-7/ADR cells than DOX (Han et al., 2015).

## TPGS-drug conjugates (TPGS prodrugs)

Polymeric modification is usually adopted to change the pharmacokinetics (PK) and pharmacodynamics (PD) of drugs and to improve the treatment efficacy. TPGS is also widely used as a matrix to form prodrug because of the benefits including self-assembly, long circulation time and improved cell uptake compared with PEGylated prodrug. It is noteworthy that after conjugation to a hydrophobic drug, the structure of the prodrug is ‘hydrophobic-hydrophilic-hydrophobic,’ and thus, the self-assembled structure must be ‘flower-like’ (which means that the PEG segment is folded, Figure 1) rather than ‘star-like’ (the typical structure of TPGS micelle). In this case, the linkage between the drug and TPGS may play an important role in the stability, as well as PK/PD, of the resultant prodrugs. For example, we synthesized two kinds of TPGS-PTX prodrug with similar structures but different linking molecules (3,3'-dithiodipropionic acid and succinic acid, denoted as TPGS-S-S-PTX and TPGS-C-C-PTX, respectively) (Bao et al., 2014). It is interesting that due to the different rigidities of the linkers, the CMC of TPGS-C-C-PTX was 3.5 times higher than that of TPGS-S-S-PTX, which greatly reduced the stability and PK properties of TPGS-C-C-PTX compared with TPGS-S-S-PTX. A similar result was obtained for the TPGS-CH = N-DOX and TPGS-DOX prodrug (Cao & Feng, 2008; Bao et al., 2016). The linker Schiff-base in TPGS-CH = N-DOX was with less softy than the succinate in TPGS-DOX. As a result, the prodrug micelles aggregated within 24 h and could not be administered by i.v. injection, while the TPGS-DOX micelles showed a *t*_1/2_ of 9.65 ± 0.94 h in rats.

### Normal TPGS-based prodrugs

Succinic acid ester, along with its analog, is probably the most used linkage between TPGS and other drugs and has been adopted to prepare TPGS-drug conjugates, such as TPGS-DOX, TPGS-cisplatin (TPGS-CPT), and TPGS-5-FU. TPGS-DOX prodrug was first synthesized by Feng’s group (Cao & Feng, 2008). This prodrug exhibited higher cellular uptake than pristine DOX and also increased the cytotoxicity in MCF-7 and C6 cells. 6.27 times effective therapeutic period, 23.6 times AUC and 5.4-fold declined heart accumulation were observed compared to free DOX. Subsequently, another type of prodrug, TPGS-CPT was reported (Mi et al., 2012). Although the cell uptake efficiency showed no difference between HepG2 hepatocarcinoma cells and SH-SY5Y neuroblastoma cells after a 2-h incubation, TPGS-CPT showed only half cytotoxicity of CPT in SH-SY5Y cells. So, they proposed that TPGS-CPT could reduce neurotoxicity in CPT chemotherapy, likely due to the neuroprotective effect of VE.

The prodrugs, such as TPGS-CPT and TPGS-5-fluorouracil (TPGS-5-FU), can be also used as a composite to form a flexible multi-drug-co-loaded system. In the subsequent work, DTX-loaded TPGS-CPT prodrug NPs were prepared with an adjustable drug ratio and showed higher *in vitro* cytotoxicity than the drugs alone at the same dose (Mi et al., 2013). TPGS-5-FU was also applied as a composite to achieve the co-delivery of 5-Fu and PTX (Wang et al., 2013; Ma et al., 2014). PTX-loaded TPGS-5-FU NPs was found to maintain the P-gp-inhibiting ability, upregulate p53 expression and induce increased cytotoxicity against 5-FU-resistant H460/TaxR cells compared to the individual drugs. This prodrug was further applied to prepare a complex nanoemulsion (NE) composed of VE and VE-PTX conjugate. Due to the same constituent unit, VE, the ‘core-matched’ NE showed a relatively high-encapsulating efficiency of >90%. *In vitro* or *in vivo* experiments revealed that this NE could overcome PTX resistance in KB-8-5 cells with reduced toxicity.

### Stimulation-responsive TPGS-based prodrugs

To achieve the greatest benefit from the P-gp inhibition ability of TPGS to effectively overcome MDR, it is best to achieve a quick separation of TPGS and the chemo-drug in the prodrug system. Thus, stimulation-responsive linkage should be a good choice. We designed a redox-responsive prodrug, TPGS-S-S-PTX. Instead of slowly-hydrolyzed succinate linker, dithiodipropionicanhydride was used to link TPGS and PTX to achieve a rapid break in the intracellular reductive environment (Bao et al., 2014). Consequently, the *in vitro* cytotoxicity of TPGS-S-S-PTX was found to be more effective than Taxol for PTX-resistant A2780/T cells due to the quick PTX release and P-gp inhibition. Improved PK and anticancer ability were achieved with fewer side effects *in vivo*. Another redox-responsive prodrug, TPGS-S-S-MTO, with the same linkage, was synthesized and also showed the ability to overcome MDR of MDA-MB-231/MDR cells both *in vitro* and *in vivo* (Qiao et al., 2017).

We also synthesized a pH-responsive prodrug, TPGS-CH = N-DOX, *via* a Schiff-based linker and prepared a stable hybrid micelles (denoted as TD) with 1,2-distearoyl-sn-glycero-3-phosphoethanolamine-N-[methoxy(poly-ethylene glycol)2000 (DSPE-PEG2000). The intracellular DOX concentration was 2.3-fold greater than that of free DOX, and the IC_50_ values were dramatically reduced by 94-fold. The tumor inhibition rate of TD was also shown to be 2.7 times that of DOX at the same dosage (5 mg DOX/kg) in MCF-7/ADR tumor model. Another pH-sensitive TPGS-DOX conjugate containing a cis-aconitate linkage was reported by Hou et al. (2016). This prodrug was used to load Ce6, a photosensitizer, and thus to obtain a chemo-photodynamic combination therapy system.

**Figure 4.**
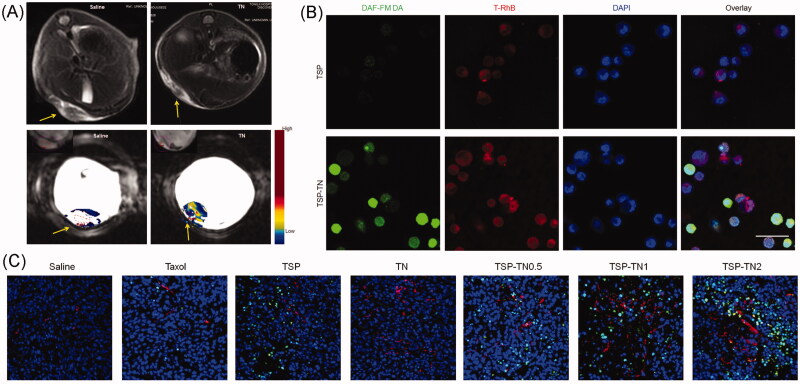
TNO_3_ and TPGS-S-S-PTX hybrid micelles. (A) *In vivo* tumor vascular permeability and blood perfusion presented by Representative MRI images in an S180 tumor model. (B) CLSM images for of micelle uptake and NO release in MCF-7/ADR cells. NO was detected by DAF-FM DA (green). Micelles were labeled by with RhB (red), and nuclei were stained by with DAPI (blue). Scale bar, 50 μm. (C) Representative immunofluorescentce images of blood vessels and tumor apoptosis of in MCF-7/ADR tumors. Blood vessels, nuclei and apoptotic cells were stained by α-CD31 antibody (red), DAPI (blue) and TUNEL (green), respectively (Yin et al., 2017b) (colour figure online).

NO regulates various pathophysiological and physiological processes, such as neurotransmission, vessel dilation, and angiogenesis (Fukumura et al., 2006). It also acts as a therapeutic agent for cancer at a relatively high concentration (Brown, 1999). Despite these advantages, the short half-life of NO is an inevitable challenge for its *in vivo* application. To achieve tumor-specific NO delivery, we synthesized a NO-releasing polymer, TPGS nitrate (TNO_3_), and then make it the enhancer for DOX (Song et al., 2014). The nitrate exhibited redox-responsive NO-releasing behavior, making it possible to achieve NO accumulation in the tumor site and also fulfill the synergistic effect of NO and DOX against tumor cells. We further chose TNO_3_ and TPGS-S-S-PTX to formulate a redox-responsive multifunctional DDS *via* the ‘core-matched’ strategy (Figure 4) (Yin et al., 2017b). Owing to the vessel dilation and angiogenesis function of NO, these mixed micelles demonstrated dramatic self-promoting drug delivery and enhanced anticancer efficiency. A significant tumor vessel density increase was observed after several treatments (almost four times in the groups without a NO donor). The increased tumor blood perfusion combined with the synergistic effect of NO and PTX resulted in significantly enhanced anticancer efficiency in S180 and MCF-7/ADR xenograft tumor model. They also prolonged the survival time in model mice and inhibited the metastasis of murine melanoma (B16F10 cells), which showed great potential for the treatment of tumors, especially in the case of MDR inhibition.

### Targeting modifications of TPGS-based prodrugs

Although the prodrug micelles displayed improved tumor accumulation compared with the free drugs through enhanced permeability and retention effect, active targeting still benefited either raising the therapeutic efficacy or reducing the side effects. In general, two strategies were used to access the targeting modifications of the TPGS-drug conjugates. One is the use of a molecule with more than two reactive groups. Typically, Anbharasi et al. (2010) synthesized a TPGS-DOX-folic acid (TPGS-DOX-FOL) prodrug *via* multiple reactions where FOL was linked with DOX *via* a short hydrazone bond. This method was a little complex and only a slight improvement (∼10%) in cell uptake efficiency was observed compared with TPGS-DOX in MCF-7 cells.

The alternative is thought to be much simpler but effective. The targeting agent was first conjugated to another amphiphilic polymer, and the conjugate is subsequently mixed with the TPGS prodrug. In our work, DSPE-PEG2000-Mal, was added to the TD to act as the foundation for the targeting modification (Bao et al., 2016). A cyclic peptide, cRGD [c(RGDfC)], with a reactive cysteine residue was coupled to the prodrug micelle *via* maleimide-thiol click chemistry as the targeting motif, and *in vitro* cell uptake and cytotoxicity were enhanced compared with the non-targeting group against the B16F10 cells. Consequently, either the tumor tissue accumulation or the therapeutic effect was significantly improved in the B16F10 xenograft tumor model. Another example is TPGS2K-FOL. Due to the same VE parts, the mixed prodrug micelles could be easily formed *via* the ‘core-matched’ mechanism, and the TPGS-CAN/TPGS-FOL and TPGS2K-MTO/TPGS2K-FOL both exhibited increased uptake and cytotoxicity, as well as enhanced tumor accumulation and anticancer capability in HT-29 and MCF-7 cells both *in vitro* and *in vivo* (Sheng et al., 2015; Guissi et al., 2017). TPGS-herceptin was also applied to form DTX-loaded TPGS-siRNA conjugate micelle system (Zhao et al., 2013a). Compared with non-targeted ones, herceptin-modified micelles showed 38.6–53.4% higher cell uptake and an ∼90% lower IC_50_ value in HER2-overexpressing SK-BR-3 cells.

## TPGS-based copolymers

There is a hydroxide group at the end of the PEG segment of TPGS. Consequently, it is easy to synthesize TPGS-based copolymers by directly inducing polymerization with/without end group modification or conjugation to other types of polymers *via* the proper linkage, just like the preparation of prodrugs. Compared with TPGS itself or TPGS-based prodrugs, the copolymers exhibited greater variability and thus could achieve diverse functions through adjustments of their composition and structure.

### TPGS-polyester copolymers

Polyester is a type of biodegradable polymer, some members of which have been approved by the FDA. Thus, TPGS-polyester copolymers have been synthesized and applied as a safe drug carrier in many situations, including TPGS-b-PLA, TPGS-b-PLGA, TPGS-b-PCL, TPGS-b-(PGA-co-PCL), TPGS-b-(PLA-co-PCL), and many anticancer drugs such as PTX, DTX, CUR, LPT, and even siRNA were encapsulated (Table S1). Compared with the free drugs, these DDSs showed enhanced cell uptake, increased anticancer efficacy both *in vitro* and *in vivo* against drug-sensitive tumor cells, and optimized PK parameters and biodistributions, such as a longer circulation time, larger AUC and greater tumor accumulation. To further improve the targeting ability of TPGS-polyester-based DDSs, a targeting modification strategy can be adopted, which is generally the same as the TPGS prodrug. Thus, it is mixed with another composite with a targeting region, for example, FOL-PEG-b-PLGA, tLyp-1-TPGS, or TPGS-transferrin (TPGS-Tf). Mei’s group used polydopamine to modify TPGS-based NPs, and then the targeting legends (aptamer or galactosamine) was conjugated to the NP surface to form the active targeting DDS (Xu et al., 2016; Zhu et al., 2016). In tumor-bearing mice, the active targeting NPs showed tumor-targeting ability (almost double that of non-targeting ones) and strong anticancer efficiency against Hela or HepG2 cells.

The most used synthesis strategy of TPGS-polyester copolymers is open ring polymerization utilizing TPGS as the macromolecular initiator with stannous octoate as a catalyst. The purity of the product is high only if the moisture is well-controlled. However, a disadvantage is the uncontrollable TPGS dissociation speed limited by the hydrolysis of the polyester. To accelerate the separation of TPGS and polyester from the same copolymer chain, we used a redox-responsive linkage of disulfide bonds to prepare a novel copolymer, TPGS-SS-PLA (Guo et al., 2016). The copolymer was then mixed with iRGD-PEG-b-PLGA to prepare a targeting NP system. Due to the receptor-mediated cellular uptake and redox-triggered drug release, the NPs showed higher PTX accumulation and cell cytotoxicity against B16F10, A2780 and A2780/T cells compared with non-targeted NPs and Taxol. The *in vivo* anticancer ability was also enhanced in S180- and B16F10-tumor bearing mice with fewer side effects.

### TPGS-polysaccharide conjugates

Polysaccharides were usually considered biocompatible, biodegradable, and weakly immunogenic, and therefore, they have been widely applied in DDS as different copolymers. We synthesized a TPGS-polysaccharide conjugate, chitosan-g-TPGS graft copolymer and prepared DOX-loaded NPs based on it (Guo et al., 2014). Due to the P-gp inhibition and reduced ATP levels, increased cytotoxicity and apoptosis were observed toward MDR MCF-7/ADR and BEL-7402/5-Fu cells compared to DOX. The PK and the anticancer activity were also superior to DOX. Su et al., (2014) reported a ROS-responsive HA-g-TPGS (TBH) graft copolymer with an arylboronic ester linker. As HA is a ligand of CD44, DOX-loaded TBH micelles can be effectively taken up by MCF-7/ADR cells *via* receptor-mediated endocytosis to achieve ROS-induced dissociation and release of the drug. The free TPGS interacts with respiratory complex II in mitochondria to generate more ROS, which accelerates the degradation of TBH and initiates a positive cycle to maintain increasing concentrations of ROS, TPGS, and DOX. TPGS also inhibits P-gp to reduce DOX efflux. Both processes accomplish the same goal of high intracellular DOX levels and clearly ultimately overcome MDR. The drug concentration in tumor tissue was found to be 5.3-fold greater and the tumor volume was clearly reduced compared with the application of free DOX.

### TPGS-based stimulation-responsive copolymers

**Figure 5.**
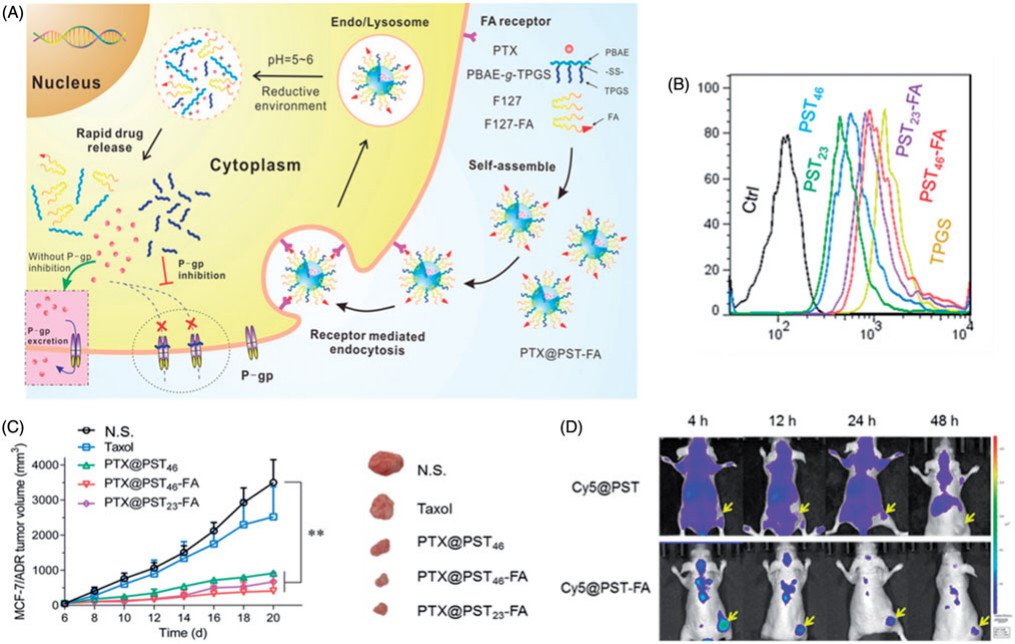
Redox/pH dual-sensitive PBAE-g-TPGS hybrid micelles. (A) Scheme of the targeting delivery and overcoming MDR. (B) Rh123 retention in MCF-7/ADR cells. (C) Live images of MCF-7/ADR tumor-bearing mice that were i.v. administered Cy5 loaded micelles. (D) *In vivo* anticancer activities (Yin et al., 2017a).

The release of drug from either TPGS-polyester or TPGS-polysaccharide DDS was limited by drug diffusion and/or the hydrolysis of the polymer matrix. To better control the drug release behavior, stimulation-responsive polymer segments can be induced to generate ‘smart’ DDS. A pH-sensitive copolymer, TPGS-b-poly(β-amino ester) (TPGS-b-PBAE), was reported by us in 2013 (Zhao et al., 2013b). The DTX-loaded TPGS-b-PBAE NPs allowed a release response in a weakly acidic environment (pH 5.5). These NPs showed increased cytotoxicity against both A2780 and MDR A2780/T cells. Because of the inhibitory properties of P-gp, a 100-fold lower IC_50_ of the NPs was observed in A2780/T cells compared with commercial DTX. Zhang et al. (2015) subsequently developed a targeting DDS based on the TPGS-b-PBAE copolymer mixed with AS1411 aptamer (Apt)-TPGS conjugate (Apt-TPGS), which could combine with nucleolin in SKOV3 ovarian cancer cells and improve endocytosis. As a benefit of the pH-triggered drug release, the PTX-loaded micelles showed improved anticancer efficiency than free PTX both *in vitro* and *in vivo*, as well as improved biosafety as a consequence of reduced myelosuppression in tumor-bearing mice. Next, the authors applied TPGS-b-PBAE to co-deliver DOX and CUR to treat human liver cancer (SMMC 7721 cells), and this strategy was found to be more effective than single-drug treatment (Zhang et al., 2017a). To achieve targeted drug delivery as well as quick dissociation of TPGS from the DDS, we further synthesized a redox/pH dual-sensitive PBAE-g-TPGS graft copolymer containing a disulfide linkage between the PBAE main chain and the TPGS side chain. (Bao et al., 2017; Yin et al., 2017a). Then it was mixed with FOL-F127 conjugate to prepare a PTX-loaded hybrid micelle system (Figure 5). As expected, the hybrid micelles demonstrated enhanced cell uptake *via* receptor-mediated endocytosis, redox/pH-responsive drug release and improved anticancer ability against MCF-7/ADR cells both i*n vivo* and *in vivo*. Mei’s group reported another pH-sensitive TPGS-b-PLGA-b-PHis) triblock copolymer as a carrier of DOX, which exhibited increased cytotoxicity (1.4–2.3- and 19.7–46.0-fold lower IC50) than normal PLGA-TPGS NPs and free DOX in MCF-7/ADR cells (Li et al., 2015).

### Other conjugates

Mehra et al., (2014) synthesized TPGS-MWCNT conjugates using amine-terminated MWCNTs and TPGS-COOH. The TPGS modification not only increased cytotoxicity and cell uptake, but also prolonged the circulation time (2.6 times *t*_1/2_) compared with MWCNTs. DOX-loaded TPGS-MWCNTs also showed the longest survival span and best anticancer efficacy *in vivo*, potentially due to the increased tumor accumulation and pH-responsive characteristics. Mei’s group conjugated TPGS onto the surface of polydopamine-coated mesoporous silica NPs (MSNs@PDA-TPGS) (Cheng et al., 2017). Compared with PEG-modified ones, MSNs@PDA-TPGS showed enhanced cellular uptake and cytotoxicity in A549 and A549/MDR cells. The *in vivo* results also revealed that MSNs-DOX@PDA-TPGS could improve DOX accumulation and dwell time at the A549/MDR tumor site and more effectively inhibit tumor growth.

## Conclusion and perspectives

In summary, TPGS-based DDS has demonstrated many superior features for the treatment of tumors. In addition to the bio-functions of TPGS itself, the synergistic effect between TPGS and therapeutic agents likely plays an important role in the final treatment efficacy. Moreover, the ‘core-matched’ strategy facilitates the preparation of various hybrid DDS based on TPGS and its modified products due to the same VE structure, which would increase the encapsulation efficiency (EE) of some drugs, precisely control their composition and achieve the targeting modification (Kutty & Feng, 2013; Ma et al., 2014; Kulhari et al., 2015; Sheng et al., 2015; Singh et al., 2016; Yin et al., 2017a,b; Zhang et al., 2017b). However, to fully understand the TPGS-based DDS and elucidate the treatment-enhancing mechanism, some uncertain phenomena must be explained. For example, some studies have shown that TPGS-containing DDS can downregulate P-gp expression (Zhang et al., 2013; Gao et al., 2014; Jin et al., 2015; Tian et al., 2015; Wang et al., 2015b,c; Liu et al., 2016a). However, as discussed above, TPGS itself only affects the function of P-gp without altering its expression. This discrepancy necessitates additional research to confirm or clarify the observed phenomenon. Another interesting result is that in some *in vitro* cases, TPGS-based DDS seemed to have much less cytotoxicity toward normal cells than toward tumor cells, which may be related to different amounts of cell uptake, especially in the presence of modifications by some targeting legends (Abbad et al., 2015; de Melo-Diogo et al., 2017, Guissi et al., 2017). Tumor cells usually internalize more particles than normal cell, leading to greater drug accumulation and thus elevated cytotoxicity. The metabolic capability and drug release speed of the cells are thought to provide another explanation, which would help to accelerate effective drug release and thus inhibit the growth of tumor cells. Moreover, the mechanisms of some bio-functions of TPGS mentioned above, including the uptake enhancer, permeation enhancer, anticancer apoptotic pathways and metastasis inhibition, were still not so clear and need further investigation.

Safety is an important feature of TPGS. Although the original preparation approved by the FDA is an oral formulation, i.v. injection of TPGS has not been reported to cause any side effects. We have also evaluated the safety of TPGS after i.v. injection in healthy Kunming mice at a dose of 200 mg/kg. (Yin et al., 2017b) The results showed that compared with saline, the TPGS group exhibited almost the same body weight changes, main organ/body index and ALT, AST, and BUN levels. H&E staining of the main organs (heart, liver, spleen, lung, and kidney) further confirmed that there were no significant differences between the two groups. TPGS was also reported to prevent hemolysis (0.32%), after being added in a positive charged dendrime, G4 PAMA (Pooja et al., 2014). However, to achieve the clinical translation, researchers should focus on several TPGS-based DDSs that are easy to be scaled-up and can be manufactured with reproducible physicochemical properties (e.g. drug loading, particle size, stability). Advanced study on safety and efficacy in clinical research is also needed. Anyway, taken together with its unique bio-functions, TPGS could serve as an effective drug carrier component that may greatly reduce ‘carrier material burden’ (Bamrungsap et al., 2012; Maksimenko et al., 2014). Thus, it could be utilized to develop a simple, but multi-functional DDS that could be applied clinically for cancer treatment.

## Supplementary Material

IDRD_Tan_et_al_Supplemental_Content.docx
