# Correction to: Tan et al., Recent developments in D-α-tocopheryl polyethylene glycol-succinate-based nanomedicine for cancer therapy

**DOI:** 10.1080/10717544.2017.1412110

**Published:** 2017-12-04

Tan S, Zou C, Zhang W, Yin M, Gao X and Tang Q. (2017). Recent developments in D-α-tocopheryl polyethylene glycol-succinate-based nanomedicine for cancer therapy. *Drug. Delivery.* VOL. 24, NO. 1, pp. 1831–1842. https://doi.org/10.1080/10717544.2017.1406561.

When the above article was first published online, affiliation for the last author “Qing Tang” was wrong.

This has now been corrected in both the online and print versions.

