# Supplementary material for: Recent developments in d-α-tocopheryl polyethylene glycol-succinate-based nanomedicine for cancer therapy
Source: Drug Deliv. 2017 Nov 28;24(1):1831–42. doi: 10.1080/10717544.2017.1406561 (PMC8241040; doi:10.1080/10717544.2017.1406561)
Supplement: IDRD_Tan_et_al_Supplemental_Content.docx [file IDRD_A_1406561_SM3481.docx]

**SUPPLEMENTAL FILE**

**Recent developments in D-α-tocopheryl polyethylene glycol-succinate-based nanomedicine for cancer therapy**

Songwei Tan^a,^*, Chenming Zou^a^, Wei Zhang^a^, Mingxing Yin^c^, Xueqin Gao^a^ & Qing Tang^a,^*

*^a^School of Pharmacy, Tongji Medical College*

*^b^Department of Integrated Chinese and Western Medicine, Union Hospital, Tongji Medical College*

*^c^Department of Pharmacy, Tongji Hospital, Tongji Medical School*

*Huazhong University of Science and Technology, Wuhan 430030, China.*

Address for correspondence: Dr. Songwei Tan, Associate Professor, School of Pharmacy, Tongji Medical College, Huazhong University of Science and Technology, 13 Hangkong Road, Wuhan 430030, PR China. Tel: +86 2783692735. E-mail: tansongwei@gmail.com. Dr. Qing Tang, Associate Professor, Department of Integrated Chinese and Western Medicine, Union Hospital, Tongji Medical College, Huazhong University of Science and Technology, 1277 Jiefang Road, Wuhan 430022, PR China. Tel: +86 2785726114. E-mail: 122022188@qq.com.

**Table S1.** List of TPGS based formulations used for cancer therapy.

| **formulations** | **composition** | **Drug** | **highlights** | **Ref** |
| --- | --- | --- | --- | --- |
| Micelles | TPGS | BAI or BAO I | Increased uptake and cytotoxicity against MCF-7 cells | ([Ma *et al.*, 2015](#_ENREF_57), [Yan *et al.*, 2015](#_ENREF_103)) |
| Micelles | TPGS2K | DOX | High EE (~85%), overcame MDR of MCF-7/ADR cells both *in vitro* and *in vivo* | ([Hao *et al.*, 2015](#_ENREF_37)) |
| Micelles | TPGS, TPGS-Cetuximab | DTX | 205.6 and 223.8 fold higher efficiency than Taxotere for the MDA-MB-468 and MDA-MB-231 treat triple negative breast cancer cell lines | ([Kutty and Feng, 2013](#_ENREF_49)) |
| Micelles | succinoyal-TPGS, TPGS-cRGD | DTX | Enhanced cytotoxicity, cellular uptake, apoptosis and anti-angiogenic comparisons of unconjugated TPGS micelles in DU145 human prostate cancer cells and Human Umblical Vein Endothelial Cells | ([Kulhari *et al.*, 2015](#_ENREF_48)) |
| Mixed micelles | TPGS, phospholipid | BAI | Increased solubility (88-fold), permeability, and relative bioavailability (533%) | ([Jin *et al.*, 2013](#_ENREF_45)) |
|  |  | API | Increased oral bioavailability (2.4 fold), increased cellular uptake, cell cytotoxicity for A549 cells, effective inhibition (3.7 fold) to S180 carcinoma mice *via* oral administration | ([Munyendo *et al.*, 2013](#_ENREF_63)) |
| Mixed micelles | TPGS, DSPE-PEG | GemC18 | Prolonged circulation time, elevated tumor concentration by 3-fold, high anticancer efficiency against human pancreatic cancer BxPC-3 xenografts | ([Wang *et al.*, 2014b](#_ENREF_99)) |
|  |  | DOX | High EE (98.2%, molar ratio of DOX/DSPE-PEG/TPGS was 1:1:0.2), enhanced *in vitro* cytotoxicity, drug uptake, apoptosis and anticancer efficacy in vivo for drug resistant H460/TaxR cancer cells | ([Jin *et al.*, 2015](#_ENREF_46)) |
|  |  | FN | Enhanced cell uptake and cytotoxicity, improved tumor inhibition rate | ([Cheng *et al.*, 2016](#_ENREF_20)) |
|  |  | BRB | 3-fold solubilization, 16-18-fold lower IC_50_s in PC3 and LNPaC micelles | ([Shen *et al.*, 2016](#_ENREF_73)) |
|  |  | SAHA and PTX | Enhanced cytotoxicity (5.9-fold) and cell migration inhibition (3.4-fold) to triple negative breast cancer (TNBC) cells | ([Kutty *et al.*, 2015](#_ENREF_50)) |
|  |  | DOX and CUR | High cytotoxicity toward MDR A549/ADR cells, prolong circulation time, high inhibiting efficiency against Lewis lung carcinoma tumor-bearing mice | ([Gu *et al.*, 2016](#_ENREF_33)) |
| Mixed micelles | TPGS, DSPE-PEG, DSPE-PEG-FOL | DTX | Small size (~20 nm), P-gp inhibition, enhanced cytotoxicity and permeation *in vitro*, higher anticancer efficacy in MDR KBv tumor model. | ([Wang *et al.*, 2015a](#_ENREF_91)) |
| Mixed micelles | TPGS, MCT,  Solutol^®^ HS-15 | TEN | 4.1-6.4-fold intestinal absorption at 0.5 h after oral administration, 5.4-fold AUC, 7.0-fold tumor accumulation (2.4 fold), | ([Zhang *et al.*, 2013b](#_ENREF_114)) |
| Mixed micelles | TPGS, MCT  Solutol^®^ HS-15, SA | TEN | Enhanced tumor accumulation, overcame MDR of MCF-7/ADR cells both *in vitro* and *in vivo* | ([Zhang *et al.*, 2013a](#_ENREF_113)) |
| Nanoemulsions | TPGS, MCT, Tween 80 | PTX | 18.8-fold lower IC_50_ in MCF-7/ADR cells, 9.4-fold tumor inhibitory rate | ([Bu *et al.*, 2014](#_ENREF_11)) |
| Mixed micelles | TPGS, Poloxamer 407 | CUR | High EE 95-86% with drug loading 1-9%, increased cellular uptake and cytotoxicity (3 fold) against drug-resistant NCl/ADR-RES cells. | ([Saxena and Hussain, 2013](#_ENREF_69)) |
| Mixed micelles | TPGS, Poloxamer 407, FOL-Poloxamer 407 | DOX | Enhanced cellular uptake, reduce drug efflux, increase cytotoxicity in SKOV3 and DOX-resistant SKOV3 cell lines, minimal toxicity to normal human cell line WRL-68 | ([Butt *et al.*, 2015](#_ENREF_12)) |
| Mixed micelles | TPGS, F127 or P84 | PTX | High tumor penetration and accumulation in B16F10 cells *in vitro* and *in vivo*, enhanced therapeutic effects in H22 xenografts tumor model | ([Cao *et al.*, 2016](#_ENREF_16)) |
| Mixed micelles | TPGS , F127 | β-Gal | Enhanced penetration in rat brain endothelial cells *via* absorptive-mediated endocytic pathway and P-gp inhibition, 3.6 fold DiR, more Rh-123 and β-Gal accumulation in rats brains | ([Meng *et al.*, 2017](#_ENREF_60)) |
| Mixed micelles | TPGS, PVPS630 | PTX | 24947-fold solubility, 4.4-fold oral bioavailability, higher anticancer efficacy in Lewis tumor-baring mice | ([Hou *et al.*, 2017](#_ENREF_38)) |
| Mixed micelles | TPGS, mPEG-PCL | RES | Increased the cell uptake and cytotoxicity against MCF-7/ADR cells | ([Wang *et al.*, 2015c](#_ENREF_96)) |
| Mixed micelles | TPGS, mPEG-PLA, CS-Sa | DTX | Enhanced oral bioavailability(2.52-fold) | ([Dou *et al.*, 2014](#_ENREF_26)) |
| Mixed micelles | TPGS, PEO-PPO-PCL | DTX | Inhibited P-gp efflux, 69-100 fold effective against MCF-7 cells | ([Shi *et al.*, 2015a](#_ENREF_75)) |
|  |  | DTX and CQ | Strongest synergism effect at DTX/CQ ratio of 0.8/0.2, higher therapeutic effects against MCF-7/ADR cells (134-195 fold smaller IC_50_) *in vitro* | ([Shi *et al.*, 2015b](#_ENREF_76)) |
| Mixed micelles | TPGS, PEOz-PLA, DSPE-PEG-FOL | DOX | pH-sensitive, increased cell uptake and cytotoxicity against KBv cells *in vitro*, targeting delivery *in vivo* | ([Zhao *et al.*, 2015](#_ENREF_119)) |
| Mixed micelles | TPGS, Soluplus^®^ | PTX | 38,000 times increased PTX solubility, increased uptake and cytotoxicity against SKOV-3, MCF-7 and MDA-MB-231 cells | ([Bernabeu *et al.*, 2016a](#_ENREF_8)) |
| Mixed micelles | TPGS2K, Tocopherol succinate | DOX | High drug loading (40wt%), high cytotoxicity, 100% long-term mice survival against CT26 and MCF-7 tumor models; same anti-cancer activity between black TOS-TPGS and free DOX. | ([Danhier *et al.*, 2014](#_ENREF_23)) |
| Mixed micelles | TPGS2K, TOS-DOX | DOX | Lamellar core structure, high drug loading (34wt%), high cytotoxicity to MCF-7 cells *in vitro* and CT26 cells *in vivo* | ([Duhem *et al.*, 2014](#_ENREF_28)) |
| Mixed micelles | TPGS, DDAB, HA | BAO I | Increased the cell uptake and cytotoxicity (2.3-fold lower IC_50_) against A549 cells, enhanced tumor growth inhibition | ([Yan *et al.*, 2017](#_ENREF_104)) |
| Mixed micelles | TPGS2K, HA-pHis | DOX | pH dependent drug release, higher and comparable cytotoxicity against MCF-7/ADR cells and MCF-7 cells, higher tumor accumulation *in vivo* | ([Qiu *et al.*, 2014](#_ENREF_67)) |
| Mixed micelles | PEG-pHis-PLGA, TPGS | GA | pH-sensitive, increased the cell cytotoxicity against MCF-7 and MCF-7/ADR cells | ([Wang *et al.*, 2015d](#_ENREF_97)) |
| Mixed micelles | TPGS, PEG-b-PDPA | DOX | pH-sensitive, increased the cell cytotoxicity (5.3-fold decreased IC_50_) against MCF-7/ADR cells, reduced heart distribution of DOX, 3.5-fold slower tumor growth | ([Shen *et al.*, 2013](#_ENREF_72)) |
| Mixed micelles | TPGS, G4 PAMA | PTX or DTX | Non-hemolysis, enhanced cytotoxicity in A549 and MCF-7 cells but reduced in normal ovary cells CHO | ([Pooja *et al.*, 2014](#_ENREF_65)) |
| Mixed micelles based nanoparticles | TPGS, P85-PEI | PTX, Twist shRNA | Down-regulation of Twist protein, 63-fold lower IC_50_ effective against 4T1 cells *in vitro*, inhibited the tumor growth and pulmonary metastasis *in vivo* | ([Shen *et al.*, 2013](#_ENREF_72)) |
|  |  | SF, surviving shRNA | Effective cell uptake and transfection *in vitro*, enhanced tumor accumulation *in vivo* against BEL-7402 cells and BEL-7402/5Fu cells | ([Shen *et al.*, 2014b](#_ENREF_71)) |
|  | TPGS, TPGS-iRGD, P85-PEI | PTX, surviving shRNA | Effective cell uptake and cytotoxicity against A549 and A549/T cells *in vitro*, overcame MDR of A549/T cells *in vivo* (1/8 tumor volume) | ([Shen *et al.*, 2014a](#_ENREF_70)) |
| Liposomes | EPC, Chol, TPGS1000-TPP | PTX | Mitochondrial targeting, mitochondrial apoptosis pathway, overcame MDR of MDR A549/cDDP both *in vitro* and *in vivo* | ([Zhou *et al.*, 2013](#_ENREF_121)) |
|  |  | SUN and/or VIN | Mitochondrial targeting, down-regulated VM channel | ([Shi *et al.*, 2015c](#_ENREF_77)) |
| Liposomes | EPC, Chol, TPGS1000-MAN, DQA-PEG2000-DSPE | PTX | Acrossing BBB, targeting vasculogenic mimicry-capable cancer cells and realizing mitochondria targeting | ([Li *et al.*, 2014](#_ENREF_52)) |
| Liposomes | TPGS, SPC, Chol, ynthetic cationic lipid, HG2C18 | PTX, LND | pH-sensitive, mitochondria targeting and overcame MDR of MCF-7/ADR both *in vitro* and *in vivo* | ([Assanhou *et al.*, 2015](#_ENREF_3)) |
| Liposomes | TPGS, egg yolk lecithin | PTX, YSV-SA | Synergistic anticancer effect of YSV and PTX against MB-231 cells both *in vitro* and *in vivo*, improved tumor-targeting | ([Jin *et al.*, 2016](#_ENREF_43)) |
|  |  | API | Synergistic anticancer effect with YSV against A549 cells both *in vitro* and *in vivo* | ([Jin *et al.*, 2017](#_ENREF_44)) |
| Solid lipid nanoparticles | TPGS, phospholipid | SIL | Down-regulated MMP-9 and Snail, inhibited migration against MDA-MB-231 cells both *in vitro* and *in vivo* | ([Xu *et al.*, 2013](#_ENREF_102)) |
|  |  | DTX | Higher relative oral bioavailability | ([Cho *et al.*, 2014](#_ENREF_21)) |
| Solid lipid nanoparticles | TPGS, N-trimethyl CS, palmitic acid, Chol | CUR | Higher oral bioavailability, and brain distribution | ([Ramalingam and Ko, 2015](#_ENREF_68)) |
| Solid lipid nanoparticles | TPGS, Brij78, glyceryl, monostearate, soya lecithin | CUR | 12 fold AUC, the relative bioavailability 942% | ([Ji *et al.*, 2016](#_ENREF_41)) |
| Solid lipid nanoparticles | TPGS, Tristearin, S-100 | RSV | Enhanced uptake and cytotoxicity against C6 glioma cell lines, 11 fold AUC, 9 fold brain distribution | ([Vijayakumar *et al.*, 2016](#_ENREF_90)) |
| SMEDDS | TPGS, Capmul, AOT ion pair complex | DOX | 4.2-fold oral bioavailability  Enhanced fibrosarcoma inhibition | ([Benival and Devarajan, 2015](#_ENREF_7)) |
| SMEDDS | TPGS, Capryol 90, Gelucire 44/14, Transcutol HP | DTX | 3.2-fold oral bioavailability  25-fold cytotoxic activity against MCF-7 cells *in vitro* | ([Valicherla *et al.*, 2016](#_ENREF_89)) |
| Nanosuspensions | TPGS | CPT | 16-fold solubility, fastly dissolving, enhanced cell uptake and cytotoxicity in MCF-7 cells *in vitro, i*mproved pharmacokinetics parameter, intratumor accumulation and anticancer efficiency | ([Tang *et al.*, 2014](#_ENREF_83)) |
|  |  | PTX | 2.4-fold LD50, increased t_1/2_, AUC and MRT, by 5-fold more effective against MDR H460/RT xenograft tumor bearing Balb/c mice | ([Gao *et al.*, 2013](#_ENREF_30), [Gao *et al.*, 2014](#_ENREF_29)) |
| Nanoparticles | TPGS | SF, CUR | Directly self-assemble, higher cytotoxicity in BEL-7402 cells and Hep G2 cells, enhanced anti-angiogenesis activities *in vitro*, 4.2- and 5.9-fold of SF and CUR concentration in tumor, strongest tumor growth inhibition in BEL-7402 cells xenograft tumor model. | ([Cao *et al.*, 2015a](#_ENREF_13)) |
| Nanoparticles | TPGS | PTX, TQR | Overcame MDR of MCF-7/ADR cells *in vitro*, decreased IL-10 concentration in the cell supernatant | ([Liu *et al.*, 2016a](#_ENREF_55)) |
| Nanoparticles | TPGS, BSA | PTX | Overcame MDR of A2780/T cells *in vitro*, 5-fold lower IC_50_ | ([Chen *et al.*, 2016](#_ENREF_17)) |
| Nanoparticles | TPGS2K, BSA | SN-38 | Overcame MDR of A549/DDP cells and A549/Taxol cells *in vitro* | ([Wang *et al.*, 2015b](#_ENREF_92)) |
| Nanoparticles | TPGS, PLGA | PTX | Enhanced cellular uptake and cytotoxicity on A549 cells *in vitro*, increased accumulation and anticancer efficacy *in vivo* | ([Wang *et al.*, 2013b](#_ENREF_94)) |
| Nanoparticles (porous) | TPGS, PLGA | DTX | 66-284-fold cytotoxicity in HeLa cells *in vitro*  Improved anti-tumor efficiency | ([Zhu *et al.*, 2014](#_ENREF_122)) |
| Nanoparticles | TPGS, HA-PBCA | MH | 1.6-fold increase in drug-loading compared to TPGS-free NPs, 2.28-fold higher cellular uptake, increased anticancer efficacy both *in vitro* (A549 cells) and *in vivo* (S180 tumor-bearing mice), selective cytotoxic effects of blank NPs against cancer cells but not normal cells | ([Abbad *et al.*, 2015](#_ENREF_1)) |
| Nanoparticles | TPGS, NaYbF_4_:Er upconversion NPs | DOX | Dual-modal probe for UCL imaging and CT, overcame the MDR of MCF-7/ARD cells. | ([Tian *et al.*, 2015](#_ENREF_88)) |
| Nanocrystals | TPGS, Cu_3_BiS_3_ nanocrystals | null | CT imaging and high-depth MSOT imaging, radio-sensitizer, photothermal agent, synergistic therapy of combined PTT and RT on BEL-7402 xenograft tumors model | ([Du *et al.*, 2017](#_ENREF_27)) |
| Nanoparticles | TPGS, nGO | null | Improved stability, high phototherapeutic effect, reduced viability of MCF-7 cells, low effect on normal human dermal fibroblasts, | ([de Melo-Diogo *et al.*, 2017](#_ENREF_25)) |
| Nanoparticles | TPGS3350, QDs | null | Nonspecific binding with MCF-7 cells. | ([Pan *et al.*, 2014](#_ENREF_64)) |
| Prodrug | TPGS-DOX | DOX | Increased cellular uptake, cell cytotoxicity for the MCF-7 cells and C6 cells. Improved pharmacokinetic and reduced heart accumulation | ([Cao and Feng, 2008](#_ENREF_14)) |
| Prodrug | TPGS-CPT | CPT | High CPT loading amount (~5 wt%) & lowered neurotoxicity | ([Mi *et al.*, 2012](#_ENREF_61)) |
| Prodrug | TPGS-GEM | GEM | Uptake without nucleoside transporter, enhanced uptake and cytotoxicity against BxPC-3 cells | ([Khare *et al.*, 2016](#_ENREF_47)) |
| Prodrug | TPGS-MTO | MTO | Cytotoxicity against MCF-7 and MCF-7/MDR cells | ([Gao *et al.*, 2016b](#_ENREF_32)) |
| Prodrug | TPGS2K-MTO, TPGS2K-FOL | MTO | pH-sensitive release, increased cellular uptake, higher cytotoxicity against MCF-7 cells but lower on hCMEC/D3 normal cells, bigger AUC, more tumor accumulation and higher anticancer ability *in vivo* | ([Guissi *et al.*, 2017](#_ENREF_34)) |
| Prodrug | TPGS-CAN, TPGS-FOL | CAN | Direct esterization between TPGS and Can, faster release at pH5.0 than pH7.4, targeting delivery and higher cytotoxicity against HT-29 cells than MCF-7 cells | ([Sheng *et al.*, 2015](#_ENREF_74)) |
| Prodrug | TPGS-DOX-FOL | DOX | Slightly improved cell uptake efficiency (~10%) and cytotoxicity against MCF-7 cells than TPGS-DOX | ([Anbharasi *et al.*, 2010](#_ENREF_2)) |
| Prodrug | TPGS-S-S-PTX | PTX | Redox-responsive, overcame MDR of A2780/T cells *in vitro*, better pharmacokinetics parameters and higher tumor accumulation than Taxol | ([Bao *et al.*, 2014](#_ENREF_5)) |
| Prodrug | TPGS-S-S-MTO | MTO | Redox-responsive, higher tumor accumulation, overcame MDR of MDA-MB-231/MDR cells both *in vitro and in vivo*, | ([Qiao *et al.*, 2017](#_ENREF_66)) |
| Prodrug | TPGS-NO_3_ | NO | Redox-responsive NO release, synergistic effect with DOX, enhanced cytotoxicity for the HepG2 cells *in vitro* and H22 cells *in vivo* | ([Song *et al.*, 2014](#_ENREF_79)) |
| Prodrug | TPGS-S-S-PTX, TPGS-NO_3_ | PTX  NO | Self-promoting drug delivery, increased tumor blood perfusion, overcame MDR of MCF-7/ADR cells both *in vitro* and *in vivo* | ([Yin *et al.*, 2017b](#_ENREF_106)) |
| Prodrug | TPGS-CH=N-DOX, DSPE-PEG2000 (with or without cRGD) | DOX | pH sensitive & overcame MDR of MCF-7/ADR cells both *in vitro* and *in vivo,* targeted H22 and B16F10 tumor by RGD modification | ([Bao *et al.*, 2016](#_ENREF_6)) |
| Prodrug | TPGS-cis-aconityl-DOX | DOX  Ce6 | pH sensitive, chemo–photodynamic combination therapy,  Enhanced therapeutic effects against A549 cells both *in vitro* and *in vivo* | ([Hou *et al.*, 2016](#_ENREF_39)) |
| Prodrug | TPGS-siPlk1, TPGS, TPGS-NH_2_, TPGS-herceptin | siPlk1, DTX | Redox-responsible siPlk1 release, synergistic effects between DTX and siPlk1, increased uptake and cytotoxicity for HER2 overexpressed SK-BR-3 cells *in vitro* | ([Zhao *et al.*, 2013a](#_ENREF_116)) |
| Prodrug &Nanoparticles | TPGS-CPT, TPGS-PLA | CPT, DTX | Co-loading CPT and DTX with variable ratio, enhanced cytotoxicity compared with single drug, and HER2 positive cancer cells targeting after Herceptin modification | ([Mi *et al.*, 2013](#_ENREF_62)) |
| Prodrug &Nanoparticles | TPGS-5-FU | 5-FU, PTX | Overcame MDR of H460/TaxR cells, TPGS-5-FU modified PTX nanoparticle to achieve synergistic effect and high cytotoxicity *in vitro* | ([Wang *et al.*, 2013a](#_ENREF_93)) |
| Prodrug & Nanoemulsions | TPGS-5-FU, VE-PTX, TPGS, VE | 5-FU, PTX | High PTX EE (95%), reversing MDR of KB-8-5 cells both *in vitro* and *in vivo* | ([Ma *et al.*, 2014](#_ENREF_58)) |
| Nanoparticles | TPGS-b-PLA | bMTSC- Platinum(II) complex | Growth inhibition on Hep-G2 cells | ([Thu *et al.*, 2013b](#_ENREF_87)) |
|  |  | CUR | High anticancer efficiency on Hep-G2 cells *in vitro* | ([Thu *et al.*, 2013a](#_ENREF_86)) |
|  |  | CRI, PAL, SIL | Enhanced cell uptake and cytotoxicity than single (Crizotinib) or dual (Crizotinib-Palbociclib) drug loaded NPs | ([de Melo-Diogo *et al.*, 2014](#_ENREF_24)) |
|  |  | DTX and TAM | Reduce antagonism of the drugs | ([Tan *et al.*, 2014](#_ENREF_82)) |
|  |  | VX680 | Enhanced cell uptake and cytotoxicity in Hela cells, 3.4 times lower IC_50_ value, phospho-histone H3 decrease at Ser10 level | ([Le *et al.*, 2016](#_ENREF_51)) |
|  |  | HS or OA | Enhanced cell uptake and cytotoxicity of NPs combination against HepG2 and HCa-F cells *in vitro*, synergistic anticancer effect *in vivo* (HCa-F cells) | ([Gao *et al.*, 2016a](#_ENREF_31)) |
| Nanoparticles | TPGS-b-PLA, DSPE-PEG | LPT | Enhanced cell uptake and cytotoxicity in MCF-7 cells, long circulation time, higher therapeutic effects against tumor bearing mice | ([Huo *et al.*, 2015](#_ENREF_40)) |
| Nanoparticles | TPGS-b-PLA, FOL-PEG-b-PLGA | DTX | Enhanced anticancer capability in MCF-7 cells both *in vitro* and *in vivo* (*i.v.* injection), long circulation time | ([Tao *et al.*, 2015](#_ENREF_85)) |
| Nanoparticles | TPGS-b-PLA, TPGS*x*-COOH, TPGS*x*-Herceptin | DTX | TPGS1000 with the best uptake and therapeutic effects against SK-BR-3 and MCF7 cells compared with TPGS2000, TPGS3350, TPGS5000 *in vitro* | ([Zhao and Feng, 2014](#_ENREF_115)) |
| Nanoparticles | TPGS-b-PLA, TPGS-Ce6, tLyp-1-TPGS | DOX | Targeted neuropilin-1 (NRP-1) receptor over pressed cell line (MCF-7/ADR), chemo-photodynamic combination therapy, specific deep penetration in tumor, overcame MDR both *in vitro* and *in vivo* | ([Jiang *et al.*, 2016](#_ENREF_42)) |
| Nanoparticles | TPGS-b-PLA, TPGS-Tf | DTX | 70- and 10-fold more cytotoxic than DTX and TPGS-b-PLA NPs against A549 cells *in vitro* | ([Singh *et al.*, 2016](#_ENREF_78)) |
| Nanoparticles | TPGS-SS-PLA, iRGD-PEG-b-PLGA | PTX | Higher uptake and cell cytotoxicity against B16F10, A2780 and A2780/T cells than non-targeted NPs and Taxol, enhanced I anticancer ability in S180- and B16F10-tumor bearing mice | ([Guo *et al.*, 2016](#_ENREF_36)) |
| Nanoparticles | TPGS-b-PLA  (star-shaped or dendritic) | PTX | Enhanced cell uptake and cytotoxicity against PC-3 or MCF-7 cells *in vitro* | ([Wang *et al.*, 2014a](#_ENREF_95), [Zeng *et al.*, 2015](#_ENREF_107)) |
| Nanoparticles | TPGS-b-PLGA | OA | Enhanced cell uptake and cytotoxicity against HepG2 cells *in vitro* | ([Bao *et al.*, 2015](#_ENREF_4)) |
|  |  | RBG | Enhanced cell uptake and cytotoxicity against HepG2 and HCa-F cells, 2-fold LD50, better pharmacodynamic property and anticancer efficacy | ([Chu *et al.*, 2016](#_ENREF_22)) |
|  |  | EMO | Enhanced cell uptake and cytotoxicity against HepG2 cells both *in vitro* and *in vivo*, liver targeting | ([Liu *et al.*, 2016b](#_ENREF_56)) |
| Nanoparticles | TPGS-b-PLGA (star-shaped) | DTX | Enhanced cell uptake and cytotoxicity than PLGA NPs and linear PLGA-b-TPGS NPs against HeLa cells *in vitro*; increased anticancer efficiency *in vivo* | ([Zeng *et al.*, 2013](#_ENREF_108)) |
|  |  | GEN | Enhanced cell uptake (~1.3 fold) and cytotoxicity (46.6 % IC_50_) compared with linear PLGA-b-TPGS NPs against HepG2 cells *in vitro* | ([Wu *et al.*, 2016](#_ENREF_101)) |
| Nanoparticles | TPGS-b-PLGA, Poloxamer 235 | DTX | Porous nanoparticles, higher antitumor efficiency than PLGA-TPGS NPs and Taxotere, overcame MDR of MCF-7/TXT cells both *in vitro* and *in vivo* | ([Tang *et al.*, 2015](#_ENREF_84)) |
| Nanoparticles | TPGS-b-PCL | PTX | High uptake and anticancer activity on MCF-7 and MDA-MB-231 cells, triple negative breast cancer cells than Abraxane or mPEG-b-PCL NPs, longer t_1/2_ than Taxol and Abraxane. | ([Bernabeu *et al.*, 2014](#_ENREF_10), [Bernabeu *et al.*, 2016b](#_ENREF_9)) |
|  |  | QUE | Increase of PCL Chain length enlarges particle size, enhanced drug loading, enhanced cell uptake and cytotoxicity om SKBR3 cells *in vitro* and the middle length chain was the best | ([Suksiriworapong *et al.*, 2016](#_ENREF_81)) |
|  |  | GEN | Enhanced cell uptake in vitro, and anticancer efficiency on Hela cells both *in vitro* and *in vivo* (*i.p.* injection) | ([Zhang *et al.*, 2015a](#_ENREF_109)) |
| Nanoparticles | TAPP-TPGS-b-PCL (star-shaped) | DTX | Phototoxicity, overcame MDR of MCF-7/ADR cells *in vitro*, or enhanced cytotoxicity on Hela cells | ([Cao *et al.*, 2015b](#_ENREF_15)), ([Wang *et al.*, 2015e](#_ENREF_98)) |
| Nanoparticles | TPGS-b-(PGA-co-PCL), PEI | TRAIL and/or endostatin genes. | Synergistic antitumor effect on Hela cells both *in vitro* and *in vivo* | ([Zheng *et al.*, 2013](#_ENREF_120)) |
| Nanoparticles | TPGS-b-(PGA-co-PCL) | HIF-1α-targeting siRNA | Efficiently deliver siRNA into CNE-2 cells, enhanced anticancer efficiency both *in vitro* and *in vivo* (*i.t.* injection) | ([Chen *et al.*, 2015](#_ENREF_18)) |
| Nanoparticles | TPGS-b-(PGA-co-PCL), CS | HIF-1α-targeting siRNA  cisplatin | Efficiently deliver siRNA into CNE-2 cells, synergistic anticancer effects | ([Lian *et al.*, 2016](#_ENREF_54)) |
| Nanoparticles | TPGS2K-b-(PGA-co-PCL) | PTX | Enhanced cell uptake in vitro, and anticancer efficiency on A-549 cells both *in vitro* and *in vivo* (*i.t.* injection) | ([Zhao *et al.*, 2013c](#_ENREF_118)) |
| Nanoparticles | TPGS-b-(PLA-co-PCL) | DTX | Enhanced cell uptake *in vitro*, and antitumor efficiency on Hela cells both *in vitro* and *in vivo* (peri-tumoral injection) | ([Wang *et al.*, 2014c](#_ENREF_100)) |
| Nanoparticles | TPGS-b-PLGA, F68 | TAN | High cytotoxicity and pro-apoptotic effects on HepG2 cells *in vitro* | ([Zhang *et al.*, 2014](#_ENREF_112)) |
| Nanoparticles | TPGS-b-PBAE, DOPC, DSPE-PEG | DTX | pH-sensitive, 100-fold lower IC_50_ in A2780/T cells | ([Zhao *et al.*, 2013b](#_ENREF_117)) |
| Mixed micelles | TPGS-b-PBAE, Apt-TPGS | DTX | pH-sensitive, targeting delivery, enhanced anticancer efficiency on SKOV3 cells both *in vitro* and *in vivo* | ([Zhang *et al.*, 2015b](#_ENREF_110)) |
| Mixed micelles | TPGS-b-PBAE | DOX  CUR | Drug co-delivery, pH-sensitive, synergistic anticancer efficiency on human liver cancer SMMC 7721 cells both *in vitro* and *in vivo* | ([Zhang *et al.*, 2017](#_ENREF_111)) |
| Mixed micelles | PBAE-g-TPGS, Fol-TPGS | PTX | pH-sensitive, targeting delivery, overcame MDR in MCF-7/ADR cells both *in vitro* and *in vivo* | ([Yin *et al.*, 2017a](#_ENREF_105)) |
| Nanoparticles | TPGS-b-PLGA-b-PLH | DOX | pH-sensitive, 2.3-fold and 48.6 lower IC_50_ than TPGS-b-PGLA and DOX against MCF-7/ADR cells, respectively | ([Li *et al.*, 2015](#_ENREF_53)) |
| Nanoparticles | CS-g-TPGS | DOX | Overcame MDR of MCF-7/ADR and BEL-7402/5-Fu cells *in vitro* | ([Guo *et al.*, 2014](#_ENREF_35)) |
| Nanoparticles | HA-g-TPGS | DOX | ROS-responsive and self-promoted drug release, Overcame MDR of MCF-7/ADR both *in vitro* and *in vivo* | ([Su *et al.*, 2014](#_ENREF_80)) |
| Nanoparticles | TPGS-MWCNT | DOX | Enhanced cell uptake *in vitro*, and anticancer efficiency on MCF-7 cells both *in vitro* and *in vivo* (*i.t.* injection) | ([Mehra *et al.*, 2014](#_ENREF_59)) |
| Nanoparticles | MSNs@PDA-TPGS | DOX | Enhanced cellular uptake and cytotoxicity on A549 and A549/ADR cells *in vitro*, increased accumulation and anticancer efficacy *in vivo* | ([Cheng *et al.*, 2017](#_ENREF_19)) |

**TABLE ABBREVIATIONS**

AOT, dox-aerosol OT; API, apigenin; BAI, baicalin; BAO I, baohuoside I; BRB, berberine; bMTSC, bis(menthone thiosemicarbazonato); CET, cetuximab; CUR, curcumin; CQ, chloroquine; Chol: cholesterol; CPT, cisplatin; CAN, cantharidin; CPT, camptothecin; CRI, crizotinib; CS, chitosan; CS-Sa, stearic acid grafted chitosan; DOX, doxorubicin; DSPE-PEG, 1,2-distearoyl-sn-glycero-3-phosphoethanolamine-N-[methoxy(poly-ethylene glycol); DTX, docetaxel; DDAB, didecyldimethylammonium bromide; DOPC, 1, 2-dioleoyl-sn-glycero-3-phosphocholine; EPC, egg phosphatidylcholine; EMO, emodin; EE, encapsulation efficiency; FN, formononetin; FOL, folate; 5-Fu, 5-Fluorouracil; GemC18, stearoyl gemcitabine; β-Gal, β-galactosidase; GA, gambogic acid; GEM, gemcitabine; GEN,genistein; HA, hyaluronic acid; HS, heparin sodium; HG2C18, 1,5-dioctadecyl-N-histidyl-l-glutamate; LND, lonidamine; LPT, lapatinib; mPEG, methylated polyethylene glycol; MCT, medium chain triglyceride; MH, morin hydrate; MTO, mitoxantrone; OA, oleanolic acid; PTX, paclitaxel; PLA, polylactic acid; PCL, poly(ε-caprolactone); PEO, polyethylene oxide; PPO, polyphenylene Oxide; PVPS630, Plasdone^®^S-630 Copovidone; PEOz-PLA, poly(2-ethyl-2-oxazoline)-PLA; PLGA, poly(lactic-co-glycolic acid); PEG-b-PDPA, PEG-b-poly(2-(diisopropylamino)ethyl methacrylate); PEI, polyetherimide; PAL, palbociclib; PGA, polyglycolic acido; PBAE, poly(β-amino ester); PLH, poly(L-histidine); QUE, quercetin; Res, resveratrol; RSV, trans-resveratrol; RBG, resibufogenin; SAHA, suberoylanilide hydroxamic acid; SA, stearylamine; Soluplus^®^, polyvinyl caprolactam-polyvinyl acetate-polyethylene glycol; SF, sorafenib; SUN, sunitinib; SPC, soybean phosphatidylcholine; SIL, silibinin; S-100, soya phosphatidylcholine; siPlk1, polo-like kinase 1siRNA; SIL, sildenafil; TEN, teniposide; TQR, tariquidar; TAM, tamoxifen; Tf, transferrin; TAN, tanshinone IIA; VIN, vinorelbine; YSV-SA, tyroservatide stearic acid conjugation.

**Reference**

Abbad, S., Wang, C., Waddad, A.Y., Lv, H. & Zhou, J., (2015). Preparation, in vitro and in vivo evaluation of polymeric nanoparticles based on hyaluronic acidpoly(butyl cyanoacrylate) and D-alpha-tocopheryl polyethylene glycol 1000 succinate for tumor-targeted delivery of morin hydrate. Int J Nanomed, 10, 305-20.

Anbharasi, V., Cao, N. & Feng, S.S., (2010). Doxorubicin conjugated to D-alpha-tocopheryl polyethylene glycol succinate and folic acid as a prodrug for targeted chemotherapy. J Biomed Mater Res A, 94A, 730-43.

Assanhou, A.G., Li, W.Y., Zhang, L., Xue, L.J., Kong, L.Y., Sun, H.B., Mo, R. & Zhang, C., (2015). Reversal of multidrug resistance by co-delivery of paclitaxel and lonidamine using a TPGS and hyaluronic acid dual-functionalized liposome for cancer treatment. Biomaterials, 73, 284-95.

Bao, X., Gao, M., Xu, H., Liu, K.-X., Zhang, C.-H., Jiang, N., Chu, Q.-C., Guan, X. & Tian, Y., (2015). A novel oleanolic acid-loaded PLGA-TPGS nanoparticle for liver cancer treatment. Drug Dev Ind Pharm, 41, 1193-203.

Bao, Y., Guo, Y., Zhuang, X., Li, D., Cheng, B., Tan, S. & Zhang, Z., (2014). D-alpha-tocopherol polyethylene glycol succinate-based redox-sensitive paclitaxel prodrug for overcoming multidrug resistance in cancer cells. Mol Pharmaceut, 11, 3196-209.

Bao, Y., Yin, M., Hu, X., Zhuang, X., Sun, Y., Guo, Y., Tan, S. & Zhang, Z., (2016). A safe, simple and efficient doxorubicin prodrug hybrid micelle for overcoming tumor multidrug resistance and targeting delivery. J Control Release, 235, 182-94.

Benival, D.M. & Devarajan, P.V., (2015). In situ lipidization as a new approach for the design of a self microemulsifying drug delivery system (SMEDDS) of doxorubicin hydrochloride for oral administration. J Biomed Nanotechnol, 11, 913-22.

Bernabeu, E., Gonzalez, L., Cagel, M., Gergic, E.P., Moretton, M.A. & Chiappetta, D.A., (2016a). Novel Soluplus (R)-TPGS mixed micelles for encapsulation of paclitaxel with enhanced in vitro cytotoxicity on breast and ovarian cancer cell lines. Colloid Surface B, 140, 403-11.

Bernabeu, E., Gonzalez, L., Legaspi, M.J., Moretton, M.A. & Chiappetta, D.A., (2016b). Paclitaxel-loaded TPGS-b-PCL nanoparticles: in vitro cytotoxicity and cellular uptake in MCF-7 and MDA-MB-231 versus mPEG-b-PCL nanoparticles and Abraxane (R). J Nanosci Nanotechno, 16, 160-70.

Bernabeu, E., Helguera, G., Legaspi, M.J., Gonzalez, L., Hocht, C., Taira, C. & Chiappetta, D.A., (2014). Paclitaxel-loaded PCL-TPGS nanoparticles: In vitro and in vivo performance compared with Abraxane (R). Colloid Surface B, 113, 43-50.

Bu, H., He, X., Zhang, Z., Yin, Q., Yu, H. & Li, Y., (2014). A TPGS-incorporating nanoemulsion of paclitaxel circumvents drug resistance in breast cancer. Int J Pharm, 471, 206-13.

Butt, A.M., Iqbal, M.C., Amin, M. & Katas, H., (2015). Synergistic effect of pH-responsive folate-functionalized poloxamer 407-TPGS-mixed micelles on targeted delivery of anticancer drugs. Int J Nanomed, 10, 1321-34.

Cao, H., Wang, Y., He, X., Zhang, Z., Yin, Q., Chen, Y., Yu, H., Huang, Y., Chen, L., Xu, M., Gu, W. & Li, Y., (2015a). Codelivery of sorafenib and curcumin by directed self-assembled nanoparticles enhances therapeutic effect on hepatocellular carcinoma. Mol Pharmaceut, 12, 922-31.

Cao, N. & Feng, S.-S., (2008). Doxorubicin conjugated to d-α-tocopheryl polyethylene glycol 1000 succinate (TPGS): Conjugation chemistry, characterization, in vitro and in vivo evaluation. Biomaterials, 29, 3856-65.

Cao, W., Zeng, X.W., Liu, G., Li, Z., Zeng, X.B., Wang, L.J., Huang, L.Q., Feng, S.S. & Mei, L., (2015b). Porphine functionalized nanoparticles of star-shaped poly(epsilon-caprolactone)-b-D-alpha-tocopheryl polyethylene glycol 1000 succinate biodegradable copolymer for chemophotodynamic therapy on cervical cancer. Acta Biomater, 26, 145-58.

Cao, X., Zhou, X., Wang, Y., Gong, T., Zhang, Z.R., Liu, R.H. & Fu, Y., (2016). Diblock- and triblock-copolymer based mixed micelles with high tumor penetration in vitro and in vivo. J Mater Chem B, 4, 3216-24.

Chen, F.C., Wu, J., Zheng, C.L., Zhu, J.B., Zhang, Y.J., You, X.R., Cai, F.J., Shah, V., Liu, J.P. & Ge, L., (2016). TPGS modified reduced bovine serum albumin nanoparticles as a lipophilic anticancer drug carrier for overcoming multidrug resistance. J Mater Chem B, 4, 3959-68.

Chen, Y., Xu, G., Zheng, Y., Yan, M., Li, Z., Zhou, Y., Mei, L. & Li, X., (2015). Nanoformulation of D-alpha-tocopheryl polyethylene glycol 1000 succinate-b-poly(epsilon-caprolactone-ran-glycolide) diblock copolymer for siRNA targeting HIF-1 alpha for nasopharyngeal carcinoma therapy. Int J Nanomed, 10, 1375-86.

Cheng, W., Liang, C., Xu, L., Liu, G., Gao, N., Tao, W., Luo, L., Zuo, Y., Wang, X., Zhang, X., Zeng, X. & Mei, L., (2017). TPGS-functionalized polydopamine-modified mesoporous silica as drug nanocarriers for enhanced lung cancer chemotherapy against multidrug resistance. Small, 13, 1700623.

Cheng, X., Yan, H., Jia, X. & Zhang, Z., (2016). Preparation and in vivo/in vitro evaluation of formononetin phospholipid/vitamin E TPGS micelles. J Drug Target, 24, 161-8.

Cho, H.J., Park, J.W., Yoon, I.S. & Kim, D.D., (2014). Surface-modified solid lipid nanoparticles for oral delivery of docetaxel: enhanced intestinal absorption and lymphatic uptake. Int J Nanomed, 9, 495-504.

Chu, Q.C., Xu, H., Gao, M., Guan, X., Liu, H.Y., Deng, S., Huo, X.K., Liu, K.X., Tian, Y. & Ma, X.C., (2016). Liver-targeting Resibufogenin-loaded poly(lactic-co-glycolic acid)-D-alpha-tocopheryl polyethylene glycol 1000 succinate nanoparticles for liver cancer therapy. Int J Nanomed, 11, 449-63.

Danhier, F., Kouhe, T.T.B., Duhem, N., Ucakar, B., Staub, A., Draoui, N., Feron, O. & Preat, V., (2014). Vitamin E-based micelles enhance the anticancer activity of doxorubicin. Int J Pharm, 476, 9-15.

De Melo-Diogo, D., Gaspar, V.M., Costa, E.C., Moreira, A.F., Oppolzer, D., Gallardo, E. & Correia, I.J., (2014). Combinatorial delivery of Crizotinib-Palbociclib-Sildenafil using TPGS-PLA micelles for improved cancer treatment. Eur J Pharm Biopharm, 88, 718-29.

De Melo-Diogo, D., Pais-Silva, C., Costa, E.C., Louro, R.O. & Correia, I.J., (2017). D-alpha-tocopheryl polyethylene glycol 1000 succinate functionalized nanographene oxide for cancer therapy. Nanomedicine, 12, 443-56.

Dou, J.F., Zhang, H.Q., Liu, X.J., Zhang, M.Y. & Zhai, G.X., (2014). Preparation and evaluation in vitro and in vivo of docetaxel loaded mixed micelles for oral administration. Colloid Surface B, 114, 20-7.

Du, J., Zheng, X., Yong, Y., Yu, J., Dong, X., Zhang, C., Zhou, R., Li, B., Yan, L., Chen, C., Gu, Z. & Zhao, Y., (2017). Design of TPGS-functionalized Cu3BiS3 nanocrystals with strong absorption in the second near-infrared window for radiation therapy enhancement. Nanoscale, 9, 8229-39.

Duhem, N., Danhier, F., Pourcelle, V., Schumers, J.M., Bertrand, O., Leduff, C.S., Hoeppener, S., Schubert, U.S., Gohy, J.F., Marchand-Brynaert, J. & Preat, V., (2014). Self-assembling doxorubicin-tocopherol succinate prodrug as a new drug delivery system: synthesis, characterization, and in vitro and in vivo anticancer activity. Bioconjugate Chem, 25, 72-81.

Gao, L., Liu, G., Ma, J., Wang, X., Wang, F., Wang, H. & Sun, J., (2014). Paclitaxel nanosuspension coated with P-gp inhibitory surfactants: II. Ability to reverse the drug-resistance of H460 human lung cancer cells. Colloid Surface B, 117, 122-7.

Gao, L., Liu, G.Y., Kang, J.R., Niu, M., Wang, Z., Wang, H.W., Ma, J.L. & Wang, X.Q., (2013). Paclitaxel nanosuspensions coated with P-gp inhibitory surfactants: I. Acute toxicity and pharmacokinetics studies. Colloid Surface B, 111, 277-81.

Gao, M., Xu, H., Bao, X., Zhang, C., Guan, X., Liu, H., Lv, L., Deng, S., Gao, D., Wang, C. & Tian, Y., (2016a). Oleanolic acid-loaded PLGA-TPGS nanoparticles combined with heparin sodium-loaded PLGA-TPGS nanoparticles for enhancing chemotherapy to liver cancer. Life Sci, 165, 63-74.

Gao, Y., Ping, Q. & Zong, L., (2016b). Preparation and antitumor activity of mitoxantrone conjugated D-alpha-tocopherylpolyethylene glycol 1000 succinate prodrug micelle. J China Pharm Univ, 47, 311-6.

Gu, Y., Li, J., Li, Y., Song, L., Li, D., Peng, L., Wan, Y. & Hua, S., (2016). Nanomicelles loaded with doxorubicin and curcumin for alleviating multidrug resistance in lung cancer. Int J Nanomed, 11, 5757-70.

Guissi, N.E.I., Li, H., Xu, Y., Semcheddine, F., Chen, M., Su, Z. & Ping, Q., (2017). Mitoxantrone- and folate-TPGS2K conjugate hybrid micellar aggregates to circumvent toxicity and enhance efficiency for breast cancer therapy. Mol Pharmaceut, 14, 1082-94.

Guo, Y., Chu, M., Tan, S., Zhao, S., Liu, H.X., Otieno, B.O., Yang, X., Xu, C. & Zhang, Z., (2014). Chitosan-g-TPGS nanoparticles for anticancer drug delivery and overcoming multidrug resistance. Mol Pharmaceut, 11, 59-70.

Guo, Y., Niu, B., Song, Q., Zhao, Y., Bao, Y., Tan, S., Si, L. & Zhang, Z., (2016). RGD-decorated redox-responsive D-alpha-tocopherol polyethylene glycol succinate-poly(lactide) nanoparticles for targeted drug delivery. J Mater Chem B, 4, 2338-50.

Hao, T.N., Chen, D.W., Liu, K.X., Qi, Y., Tian, Y., Sun, P.Y., Liu, Y.H. & Li, Z., (2015). Micelles of d-alpha-tocopheryl polyethylene glycol 2000 succinate (TPGS 2k) for doxorubicin delivery with reversal of multidrug resistance. ACS Appl Mater Inter, 7, 18064-75.

Hou, J., Sun, E., Zhang, Z.-H., Wang, J., Yang, L., Cui, L., Ke, Z.-C., Tan, X.-B., Jia, X.-B. & Lv, H., (2017). Improved oral absorption and anti-lung cancer activity of paclitaxel-loaded mixed micelles. Drug deliv, 24, 261-9.

Hou, W.X., Zhao, X., Qian, X.Q., Pan, F., Zhang, C.L., Yang, Y.M., De La Fuente, J.M. & Cui, D.X., (2016). pH-sensitive self-assembling nanoparticles for tumor near-infrared fluorescence imaging and chemo-photodynamic combination therapy. Nanoscale, 8, 104-16.

Huo, Z.J., Wang, S.J., Wang, Z.Q., Zuo, W.S., Liu, P., Pang, B. & Liu, K., (2015). Novel nanosystem to enhance the antitumor activity of lapatinib in breast cancer treatment: Therapeutic efficacy evaluation. Cancer Sci, 106, 1429-37.

Ji, H.Y., Tang, J.L., Li, M.T., Ren, J.M., Zheng, N.N. & Wu, L.H., (2016). Curcumin-loaded solid lipid nanoparticles with Brij78 and TPGS improved in vivo oral bioavailability and in situ intestinal absorption of curcumin. Drug deliv, 23, 459-70.

Jiang, D., Gao, X.L., Kang, T., Feng, X.Y., Yao, J.H., Yang, M.S., Jing, Y.X., Zhu, Q.Q., Feng, J.X. & Chen, J., (2016). Actively targeting D-alpha-tocopheryl polyethylene glycol 1000 succinate-poly(lactic acid) nanoparticles as vesicles for chemo-photodynamic combination therapy of doxorubicin-resistant breast cancer. Nanoscale, 8, 3100-18.

Jin, X., Li, M., Yin, L., Zhou, J., Zhang, Z. & Lv, H., (2016). Tyroservatide-TPGS-Paclitaxel liposomes: Tyroservatide as a targeting ligand for improving breast cancer treatment. Nanomedicine : nanotechnology, biology, and medicine, 13, 1105-15.

Jin, X., Yang, Q. & Zhang, Y., (2017). Synergistic apoptotic effects of apigenin TPGS liposomes and tyroservatide: implications for effective treatment of lung cancer. Int J Nanomed, 12, 5109-18.

Jin, X., Zhang, Z.H., Sun, E., Tan, X.B., Zhu, F.X. & Jia, X.B., (2013). A novel drug-phospholipid complex loaded micelle for baohuoside I enhanced oral absorption: in vivo and in vitro evaluations. Drug Dev Ind Pharm, 39, 1421-30.

Jin, Y., Zhang, Z., Zhao, T., Liu, X. & Jian, L., (2015). Mixed micelles of doxorubicin overcome multidrug resistance by inhibiting the expression of p-glycoprotein. J Biomed Nanotechnol, 11, 1330-8.

Khare, V., Al Sakarchi, W., Gupta, P.N., Curtis, A.D.M. & Hoskins, C., (2016). Synthesis and characterization of TPGS-gemcitabine prodrug micelles for pancreatic cancer therapy. Rsc Adv, 6, 60126-37.

Kulhari, H., Pooja, D., Shrivastava, S., Telukutala, S.R., Barui, A.K., Patra, C.R., Vegi, G.M.N., Adams, D.J. & Sistla, R., (2015). Cyclic-RGDfK peptide conjugated succinoyl-TPGS nanomicelles for targeted delivery of docetaxel to integrin receptor over-expressing angiogenic tumours. Nanomed-Nanotechnol, 11, 1511-20.

Kutty, R.V. & Feng, S.S., (2013). Cetuximab conjugated vitamin E TPGS micelles for targeted delivery of docetaxel for treatment of triple negative breast cancers. Biomaterials, 34, 10160-71.

Kutty, R.V., Tay, C.Y., Lim, C.S., Feng, S.S. & Leong, D.T., (2015). Anti-migratory and increased cytotoxic effects of novel dual drug-loaded complex hybrid micelles in triple negative breast cancer cells. Nano Res, 8, 2533-47.

Le, T.T.D., Ha, P.T., Tran, T.H.Y., Nguyen, D.T., Nguyen, H.N., Bui, V.K. & Hoang, M.N., (2016). In vitro evaluation of Aurora kinase inhibitor-VX680-in formulation of PLA-TPGS nanoparticles. Adv Nat Sci-Nanosci, 7, 025010.

Li, X.-Y., Zhao, Y., Sun, M.-G., Shi, J.-F., Ju, R.-J., Zhang, C.-X., Li, X.-T., Zhao, W.-Y., Mu, L.-M., Zeng, F., Lou, J.-N. & Lu, W.-L., (2014). Multifunctional liposomes loaded with paclitaxel and artemether for treatment of invasive brain glioma. Biomaterials, 35, 5591-604.

Li, Z., Qiu, L., Chen, Q., Hao, T., Qiao, M., Zhao, H., Zhang, J., Hu, H., Zhao, X., Chen, D. & Mei, L., (2015). pH-sensitive nanoparticles of poly(L-histidine)-poly(lactide-co-glycolide)-tocopheryl polyethylene glycol succinate for anti-tumor drug delivery. Acta Biomater, 11, 137-50.

Lian, D.Z., Chen, Y.H., Xu, G., Zeng, X.W., Li, Z.L., Li, Z.H., Zhou, Y.Y., Mei, L. & Li, X.M., (2016). Delivery of siRNA targeting HIF-1 alpha loaded chitosan modified D-alpha-tocopheryl polyethylene glycol 1000 succinate-b-poly(epsilon-caprolactone-ran-glycolide) nanoparticles into nasopharyngeal carcinoma cell to improve the therapeutic efficacy of cisplatin. Rsc Adv, 6, 37740-9.

Liu, B.Y., Wu, C., He, X.Y., Zhuo, R.X. & Cheng, S.X., (2016a). Multi-drug loaded vitamin E-TPGS nanoparticles for synergistic drug delivery to overcome drug resistance in tumor treatment. Sci Bull, 61, 552-60.

Liu, H.Y., Gao, M., Xu, H., Guan, X., Lv, L., Deng, S., Zhang, C.H. & Tian, Y., (2016b). A promising emodin-loaded poly (lactic-co-glycolic acid)-d-alpha-tocopheryl polyethylene glycol 1000 succinate nanoparticles for liver cancer therapy. Pharm Res, 33, 217-36.

Ma, W., Wang, J. & Tu, P., (2015). Preparation and characterization of baicalin-loaded polymeric micelles and its inhibition on MCF-7 cells. Chin Tradit Herbal Drugs, 46, 507-12.

Ma, Y., Liu, D., Wang, D., Wang, Y., Fu, Q., Fallon, J.K., Yang, X., He, Z. & Liu, F., (2014). Combinational delivery of hydrophobic and hydrophilic anticancer drugs in single nanoemulsions to treat MDR in cancer. Mol Pharmaceut, 11, 2623-30.

Mehra, N.K., Verma, A.K., Mishra, P.R. & Jain, N.K., (2014). The cancer targeting potential of d-α-tocopheryl polyethylene glycol 1000 succinate tethered multi walled carbon nanotubes. Biomaterials, 35, 4573-88.

Meng, X., Liu, J., Yu, X., Li, J., Lu, X. & Shen, T., (2017). Pluronic f127 and d-alpha-tocopheryl polyethylene glycol succinate (TPGS) mixed micelles for targeting drug delivery across the blood brain barrier. Sci Rep-UK, 7, 2964.

Mi, Y., Zhao, J. & Feng, S.-S., (2012). Vitamin E TPGS prodrug micelles for hydrophilic drug delivery with neuroprotective effects. Int J Pharm, 438, 98-106.

Mi, Y., Zhao, J. & Feng, S.S., (2013). Targeted co-delivery of docetaxel, cisplatin and herceptin by vitamin E TPGS-cisplatin prodrug nanoparticles for multimodality treatment of cancer. J Control Release, 169, 185-92.

Munyendo, W.L.L., Zhang, Z.H., Abbad, S., Waddad, A.Y., Lv, H.X., Baraza, L.D. & Zhou, J.P., (2013). Micelles of TPGS modified apigenin phospholipid complex for oral administration: Preparation, in vitro and in vivo evaluation. J Biomed Nanotechnol, 9, 2034-47.

Pan, J., Wan, D., Bian, Y., Guo, Y., Jin, F., Wang, T. & Gong, J., (2014). Reduction of nonspecific binding for cellular imaging using quantum dots conjugated with vitamin E. Aiche J, 60, 1591-7.

Pooja, D., Kulhari, H., Singh, M.K., Mukherjee, S., Rachamalla, S.S. & Sistla, R., (2014). Dendrimer-TPGS mixed micelles for enhanced solubility and cellular toxicity of taxanes. Colloid Surface B, 121, 461-8.

Qiao, H., Zhu, Z., Fang, D., Sun, Y., Kang, C., Di, L., Zhang, L. & Gao, Y., (2017). Redox-triggered mitoxantrone prodrug micelles for overcoming multidrug-resistant breast cancer. J Drug Target, 1-11.

Qiu, L., Qiao, M., Chen, Q., Tian, C., Long, M., Wang, M., Li, Z., Hu, W., Li, G., Cheng, L., Cheng, L., Hu, H., Zhao, X. & Chen, D., (2014). Enhanced effect of pH-sensitive mixed copolymer micelles for overcoming multidrug resistance of doxorubicin. Biomaterials, 35, 9877-87.

Ramalingam, P. & Ko, Y.T., (2015). Enhanced oral delivery of curcumin from N-trimethyl chitosan surface-modified solid lipid nanoparticles: pharmacokinetic and brain distribution evaluations. Pharm Res, 32, 389-402.

Saxena, V. & Hussain, M.D., (2013). Polymeric mixed micelles for delivery of curcumin to multidrug resistant ovarian cancer. J Biomed Nanotechnol, 9, 1146-54.

Shen, J., Meng, Q., Sui, H., Yin, Q., Zhang, Z., Yu, H. & Li, Y., (2014a). iRGD conjugated TPGS mediates codelivery of Paclitaxel and Survivin shRNA for the reversal of Lung cancer resistance. Mol Pharmaceut, 11, 2579-91.

Shen, J., Sun, H., Meng, Q., Yin, Q., Zhang, Z., Yu, H. & Li, Y., (2014b). Simultaneous inhibition of tumor growth and angiogenesis for resistant hepatocellular carcinoma by co-delivery of sorafenib and Survivin small hairpin RNA. Mol Pharmaceut, 11, 3342-51.

Shen, J., Sun, H., Xu, P., Yin, Q., Zhang, Z., Wang, S., Yu, H. & Li, Y., (2013). Simultaneous inhibition of metastasis and growth of breast cancer by co-delivery of twist shRNA and paclitaxel using pluronic P85-PEI/TPGS complex nanoparticles. Biomaterials, 34, 1581-90.

Shen, R., Kim, J.J., Yao, M.Y. & Elbayoumi, T.A., (2016). Development and evaluation of vitamin E D-alpha-tocopheryl polyethylene glycol 1000 succinate-mixed polymeric phospholipid micelles of berberine as an anticancer nanopharmaceutical. Int J Nanomed, 11, 1687-700.

Sheng, S.H., Zhang, T., Li, S.J., Wei, J., Xu, G.J., Sun, T.H., Chen, Y.H., Lu, F.Q., Li, Y.C., Yang, J.H., Yu, H.Q., Liu, T.J. & Han, G., (2015). Targeting vitamin E TPGS-cantharidin conjugate nanoparticles for colorectal cancer therapy. Rsc Adv, 5, 53846-56.

Shi, C., Zhang, Z., Wang, F., Ji, X., Zhao, Z. & Luan, Y., (2015a). Docetaxel-loaded PEO-PPO-PCL/TPGS mixed micelles for overcoming multidrug resistance and enhancing antitumor efficacy. J Mater Chem B, 3, 4259-71.

Shi, C.H., Zhang, Z.Q., Shi, J.X., Wang, F. & Luan, Y.X., (2015b). Co-delivery of docetaxel and chloroquine via PEO-PPO-PCL/TPGS micelles for overcoming multidrug resistance. Int J Pharm, 495, 932-9.

Shi, J.F., Sun, M.G., Li, X.Y., Zhao, Y., Ju, R.J., Mu, L.M., Yan, Y., Li, X.T., Zeng, F. & Lu, W.L., (2015c). A combination of targeted sunitinib liposomes and targeted vinorelbine liposomes for treating invasive breast cancer. J Biomed Nanotechnol, 11, 1568-82.

Singh, R.P., Sharma, G., Sonali, Agrawal, P., Pandey, B.L., Koch, B. & Muthu, M.S., (2016). Transferrin receptor targeted PLA-TPGS micelles improved efficacy and safety in docetaxel delivery. Int J Bio Macromol, 83, 335-44.

Song, Q., Tan, S., Zhuang, X., Guo, Y., Zhao, Y., Wu, T., Ye, Q., Si, L. & Zhang, Z., (2014). Nitric oxide releasing D-alpha-tocopheryl polyethylene glycol succinate for enhancing antitumor activity of doxorubicin. Mol Pharmaceut, 11, 4118-29.

Su, Z., Chen, M., Xiao, Y., Sun, M., Zong, L., Asghar, S., Dong, M., Li, H., Ping, Q. & Zhang, C., (2014). ROS-triggered and regenerating anticancer nanosystem: An effective strategy to subdue tumor's multidrug resistance. J Control Release, 196, 370-83.

Suksiriworapong, J., Phoca, K., Ngamsom, S., Sripha, K., Moongkarndi, P. & Junyaprasert, V.B., (2016). Comparison of poly(epsilon-caprolactone) chain lengths of poly(epsilon-caprolactone)-co-d-alpha-tocopheryl-poly(ethylene glycol) 1000 succinate nanoparticles for enhancement of quercetin delivery to SKBR3 breast cancer cells. Eur J Pharm Biopharm, 101, 15-24.

Tan, G.-R., Feng, S.-S. & Leong, D.T., (2014). The reduction of anti-cancer drug antagonism by the spatial protection of drugs with PLA-TPGS nanoparticles. Biomaterials, 35, 3044-51.

Tang, X.J., Han, M., Yang, B., Shen, Y.Q., He, Z.G., Xu, D.H. & Gao, J.Q., (2014). Nanocarrier improves the bioavailability, stability and antitumor activity of camptothecin. Int J Pharm, 477, 536-45.

Tang, X.L., Liang, Y., Feng, X.J., Zhang, R.B., Jin, X. & Sun, L.L., (2015). Co-delivery of docetaxel and Poloxamer 235 by PLGA-TPGS nanoparticles for breast cancer treatment. Mat Sci Eng C-Mater, 49, 348-55.

Tao, W., Zhang, J., Zeng, X., Liu, D., Liu, G., Zhu, X., Liu, Y., Yu, Q., Huang, L. & Mei, L., (2015). Blended nanoparticle system based on miscible structurally similar polymers: A safe, simple, targeted, and surprisingly high efficiency vehicle for cancer therapy. Adv Healthc Mater, 4, 1203-14.

Thu, H.P., Quang, D.T., Trang, M.T.T., Ha, T.T.H., Nam, N.H., Phuc, N.X., Nguyet, T.T.M., Thong, P.Q., Tuyet, P.T.H., Oanh, V.T.K. & Huong, L.M., (2013a). In vitro apoptosis enhancement of Hep-G2 cells by PLA-TPGS and PLA-PEG block copolymer encapsulated curcumin nanoparticles. Chem Lett, 42, 255-7.

Thu, H.P., Tuyet, P.T.H., Trang, M.T.T., Nam, N.H., Hieu, T.T.N., Quang, L.Q., Hang, T.T.N., Ha, T.T.H., Nghi, D.H., Cuong, L.H. & Huong, L.M., (2013b). Preparation and biological properties of platinum(II) complex-loaded copolymer PLA-TPGS. J Nanomater, 12, 1-9.

Tian, G., Zheng, X., Zhang, X., Yin, W., Yu, J., Wang, D., Zhang, Z., Yang, X., Gu, Z. & Zhao, Y., (2015). TPGS-stabilized NaYbF4:Er upconversion nanoparticles for dual-modal fluorescent/CT imaging and anticancer drug delivery to overcome multi-drug resistance. Biomaterials, 40, 107-16.

Valicherla, G.R., Dave, K.M., Syed, A.A., Riyazuddin, M., Gupta, A.P., Singh, A., Wahajuddin, Mitra, K., Datta, D. & Gayen, J.R., (2016). Formulation optimization of Docetaxel loaded self-emulsifying drug delivery system to enhance bioavailability and anti-tumor activity. Sci Rep-UK, 6, 26895.

Vijayakumar, M.R., Kumari, L., Patel, K.K., Vuddanda, P.R., Vajanthri, K.Y., Mahto, S.K. & Singh, S., (2016). Intravenous administration of trans-resveratrol-loaded TPGS-coated solid lipid nanoparticles for prolonged systemic circulation, passive brain targeting and improved in vitro cytotoxicity against C6 glioma cell lines. Rsc Adv, 6, 50336-48.

Wang, A.-T., Liang, D.-S., Liu, Y.-J. & Qi, X.-R., (2015a). Roles of ligand and TPGS of micelles in regulating internalization, penetration and accumulation against sensitive or resistant tumor and therapy for multidrug resistant tumors. Biomaterials, 53, 160-72.

Wang, D.-F., Rong, W.-T., Lu, Y., Hou, J., Qi, S.-S., Xiao, Q., Zhang, J., You, J., Yu, S.-Q. & Xu, Q., (2015b). TPGS(2k)/PLGA nanoparticles for overcoming multidrug resistance by interfering mitochondria of human alveolar adenocarcinoma cells. ACS Appl Mater Inter, 7, 3888-901.

Wang, D., Tang, J.L., Wang, Y.J., Ramishetti, S., Fu, Q., Racette, K. & Liu, F., (2013a). Multifunctional nanoparticles based on a single-molecule modification for the treatment of drug-resistant cancer. Mol Pharmaceut, 10, 1465-9.

Wang, G.Y., Yu, B., Wu, Y.Q., Huang, B.L., Yuan, Y. & Liu, C.S., (2013b). Controlled preparation and antitumor efficacy of vitamin E TPGS-functionalized PLGA nanoparticles for delivery of paclitaxel. Int J Pharm, 446, 24-33.

Wang, H., Zhang, C., Zhang, L., Liu, L., Zheng, Y. & Zhu, D., (2014a). Synthesis of nanoparticles of star-shaped mannitol-core PLA-TPGS copolymer for delivery of paclitaxel and activity of anti-prostate cancer. Chem J Chinese U, 35, 2239-45.

Wang, S., Chen, R., Morott, J., Repka, M.A., Wang, Y. & Chen, M., (2015c). mPEG-b-PCL/TPGS mixed micelles for delivery of resveratrol in overcoming resistant breast cancer. Expert Opin Drug Deliv, 12, 361-73.

Wang, S.P., Yang, Y., Wang, Y.T. & Chen, M.W., (2015d). Gambogic acid-loaded pH-sensitive mixed micelles for overcoming breast cancer resistance. Int J Pharm, 495, 840-8.

Wang, T., Zhu, D., Liu, G., Tao, W., Cao, W., Zhang, L., Wang, L., Chen, H., Mei, L., Huang, L. & Zeng, X., (2015e). DTX-loaded star-shaped TAPP-PLA-b-TPGS nanoparticles for cancer chemical and photodynamic combination therapy. Rsc Adv, 5, 50617-27.

Wang, Y.Z., Fan, W., Dai, X., Katragadda, U., Mckinley, D., Teng, Q. & Tan, C., (2014b). Enhanced tumor delivery of gemcitabine via PEG-DSPE/TPGS mixed micelles. Mol Pharmaceut, 11, 1140-50.

Wang, Z., Wu, Y., Zeng, X., Ma, Y., Liu, J., Tang, X., Gao, Y., Liu, K., Zhang, J., Ming, P., Huang, L. & Mei, L., (2014c). Antitumor efficiency of D-alpha -tocopheryl polyethylene glycol 1000 succinate-b-poly(epsilon-caprolactone-ran-lactide) nanoparticle-based delivery of docetaxel in mice bearing cervical cancer. J Biomed Nanotechnol, 10, 1509-19.

Wu, B.Q., Liang, Y., Tan, Y., Xie, C.M., Shen, J., Zhang, M., Liu, X.K., Yang, L.X., Zhang, F.J., Liu, L., Cai, S.Y., Huai, D., Zheng, D.H., Zhang, R.B., Zhang, C., Chen, K., Tang, X.L. & Sui, X.M., (2016). Genistein-loaded nanoparticles of star-shaped diblock copolymer mannitol-core PLGA-TPGS for the treatment of liver cancer. Mat Sci Eng C-Mater, 59, 792-800.

Xu, P., Yin, Q., Shen, J., Chen, L., Yu, H., Zhang, Z. & Li, Y., (2013). Synergistic inhibition of breast cancer metastasis by silibinin-loaded lipid nanoparticles containing TPGS. Int J Pharm, 454, 21-30.

Yan, H., Jia, X., Zhang, Z., Sun, E. & Song, J., (2015). Effect of D-alpha-tocopherol polyethylene glycol 1000 succinate on inhibition of MCF-7 cell proliferation by baohuoside I. Chin Tradit Herbal Drugs, 46, 384-8.

Yan, H., Song, J., Jia, X. & Zhang, Z., (2017). Hyaluronic acid-modified didecyldimethylammonium bromide/d-a-tocopheryl polyethylene glycol succinate mixed micelles for delivery of baohuoside I against non-small cell lung cancer: in vitro and in vivo evaluation. Drug deliv, 24, 30-9.

Yin, M., Bao, Y., Gao, X., Wu, Y., Sun, Y., Zhao, X., Xu, H., Zhang, Z. & Tan, S., (2017a). Redox/pH dual-sensitive hybrid micelles for targeting delivery and overcoming multidrug resistance of cancer. J Mater Chem B, 5, 2964-78.

Yin, M., Tan, S., Bao, Y. & Zhang, Z., (2017b). Enhanced tumor therapy via drug co-delivery and in situ vascular-promoting strategy. J Control Release, 258, 108-20.

Zeng, X., Tao, W., Wang, Z., Zhang, X., Zhu, H., Wu, Y., Gao, Y., Liu, K., Jiang, Y., Huang, L., Mei, L. & Feng, S.-S., (2015). Docetaxel-Loaded nanoparticles of dendritic amphiphilic block copolymer H40-PLA-b-TPGS for cancer treatment. Part Part Syst Char, 32, 112-22.

Zeng, X.W., Tao, W., Mei, L., Huang, L.G., Tan, C.Y. & Feng, S.S., (2013). Cholic acid-functionalized nanoparticles of star-shaped PLGA-vitamin E TPGS copolymer for docetaxel delivery to cervical cancer. Biomaterials, 34, 6058-67.

Zhang, H.L., Liu, G., Zeng, X.W., Wu, Y.P., Yang, C.M., Mei, L., Wang, Z.Y. & Huang, L.Q., (2015a). Fabrication of genistein-loaded biodegradable TPGS-b-PCL nanoparticles for improved therapeutic effects in cervical cancer cells. Int J Nanomed, 10, 2461-73.

Zhang, J., Chen, R., Fang, X., Chen, F., Wang, Y. & Chen, M., (2015b). Nucleolin targeting AS1411 aptamer modified pH-sensitive micelles for enhanced delivery and antitumor efficacy of paclitaxel. Nano Res, 8, 201-18.

Zhang, J., Li, J., Shi, Z., Yang, Y., Xie, X., Lee, S.M., Wang, Y., Leong, K.W. & Chen, M., (2017). pH-sensitive polymeric nanoparticles for co-delivery of doxorubicin and curcumin to treat cancer via enhanced pro-apoptotic and anti-angiogenic activities. Acta Biomater, 58, 349-64.

Zhang, J., Li, Y., Fang, X., Zhou, D., Wang, Y. & Chen, M., (2014). TPGS-g-PLGA/Pluronic F68 mixed micelles for tanshinone IIA delivery in cancer therapy. Int J Pharm, 476, 185-98.

Zhang, Z., Liu, Z., Ma, L., Jiang, S., Wang, Y., Yu, H., Yin, Q., Cui, J. & Li, Y., (2013a). Reversal of multidrug resistance by mitochondrial targeted self-assembled nanocarrier based on stearylamine. Mol Pharmaceut, 10, 2426-34.

Zhang, Z., Ma, L., Jiang, S., Liu, Z., Huang, J., Chen, L., Yu, H. & Li, Y., (2013b). A self-assembled nanocarrier loading teniposide improves the oral delivery and drug concentration in tumor. J Control Release, 166, 30-7.

Zhao, J. & Feng, S.-S., (2014). Effects of PEG tethering chain length of vitamin E TPGS with a Herceptin-functionalized nanoparticle formulation for targeted delivery of anticancer drugs. Biomaterials, 35, 3340-7.

Zhao, J., Mi, Y. & Feng, S.S., (2013a). Targeted co-delivery of docetaxel and siPlk1 by herceptin-conjugated vitamin E TPGS based immunomicelles. Biomaterials, 34, 3411-21.

Zhao, S., Tan, S., Guo, Y., Huang, J., Chu, M., Liu, H. & Zhang, Z., (2013b). pH-sensitive docetaxel-loadedd-α-tocopheryl polyethylene glycol succinate–poly(β-amino ester) copolymer nanoparticles for overcoming multidrug resistance. Biomacromolecules, 14, 2636-46.

Zhao, T.J., Chen, H.Z., Dong, Y.C., Zhang, J.J., Huang, H.D., Zhu, J. & Zhang, W., (2013c). Paclitaxel-loaded poly(glycolide-co-epsilon-caprolactone)-b-D-alpha-tocopheryl polyethylene glycol 2000 succinate nanoparticles for lung cancer therapy. Int J Nanomed, 8, 1947-57.

Zhao, Y., Zhou, Y., Wang, D., Gao, Y., Li, J., Ma, S., Zhao, L., Zhang, C., Liu, Y. & Li, X., (2015). pH-responsive polymeric micelles based on poly(2-ethyl-2-oxazoline)-poly(d,l-lactide) for tumor-targeting and controlled delivery of doxorubicin and P-glycoprotein inhibitor. Acta Biomater, 17, 182-92.

Zheng, Y., Chen, H.B., Zeng, X.W., Liu, Z.G., Xiao, X.J., Zhu, Y.Q., Gu, D.Y. & Mei, L., (2013). Surface modification of TPGS-b-(PCL-ran-PGA) nanoparticles with polyethyleneimine as a co-delivery system of TRAIL and endostatin for cervical cancer gene therapy. Nanoscale Res Lett, 8, 161.

Zhou, J., Zhao, W.Y., Ma, X., Ju, R.J., Li, X.Y., Li, N., Sun, M.G., Shi, J.F., Zhang, C.X. & Lu, W.L., (2013). The anticancer efficacy of paclitaxel liposomes modified with mitochondrial targeting conjugate in resistant lung cancer. Biomaterials, 34, 3626-38.

Zhu, H., Chen, H., Zeng, X., Wang, Z., Zhang, X., Wu, Y., Gao, Y., Zhang, J., Liu, K., Liu, R., Cai, L., Mei, L. & Feng, S.-S., (2014). Co-delivery of chemotherapeutic drugs with vitamin E TPGS by porous PLGA nanoparticles for enhanced chemotherapy against multi-drug resistance. Biomaterials, 35, 2391-400.
